# Postsynaptic lncRNA *Sera*/Pkm2 pathway orchestrates the transition from social competition to rank by remodeling the neural ensemble in mPFC

**DOI:** 10.1038/s41421-024-00706-8

**Published:** 2024-08-20

**Authors:** Ling-Shuang Zhu, Chuan Lai, Chao-Wen Zhou, Hui-Yang Chen, Zhi-Qiang Liu, Ziyuan Guo, Hengye Man, Hui-Yun Du, Youming Lu, Feng Hu, Zhiye Chen, Kai Shu, Ling-Qiang Zhu, Dan Liu

**Affiliations:** 1 Department of Pathophysiology, Key Lab of Neurological Disorder of Education Ministry, School of Basic Medicine, Tongji Medical College, Huazhong University of Science and Technology, Wuhan, Hubei China; 2 Center for Stem Cell and Organoid Medicine (CuSTOM), Division of Developmental Biology, Cincinnati Children’s Hospital Medical Center, Cincinnati, OH USA; 3 Department of Biology, Boston University, Boston, MA USA; 4 Department of Neurosurgery, Tongji Hospital, Tongji Medical College, Huazhong University of Science and Technology, Wuhan, Hubei China

**Keywords:** Non-coding RNAs, Membrane trafficking

## Abstract

Individuals’ continuous success in competitive interactions with conspecifics strongly affects their social hierarchy. Medial prefrontal cortex (mPFC) is the key brain region mediating both social competition and hierarchy. However, the molecular regulatory mechanisms underlying the neural ensemble in the mPFC remains unclear. Here, we demonstrate that in excitatory neurons of prelimbic cortex (PL), lncRNA *Sera* remodels the utilization of *Pkm* Exon9 and Exon10, resulting in a decrease in the Pkm1/2 ratio in highly competitive mice. By employing a tet-on/off system, we disrupt or rebuild the normal Pkm1/2 ratio by controlling the expression of Pkm2 in PL excitatory neurons. We find that long-term Pkm2 modulation induces timely competition alteration and hysteretic rank change, through phosphorylating the Ser845 site of GluA1. Together, this study uncovers a crucial role of lncRNA *Sera*/Pkm2 pathway in the transition of social competition to rank by remodeling neural ensemble in mPFC.

## Introduction

Most social animals self-organize into social hierarchies to allocate a strict priority order in dividing available resources among the individuals^1^. After establishment, hierarchies are typically maintained in a long-term period, which decreases aggression, reduces injury risks, promotes survival, and conserves energy for the whole group^2,3^. Despite its relative stability, social hierarchy still faces reestablishment challenge, because individual behavior is highly plastic in response to differences in environment, ecology or its own internal state^4,5^. Individuals’ success to win competitive interactions with conspecifics has been implicated as the most straightforward way to establish dominance^6,7^. However, the mechanisms underlying how competition success determines social hierarchy remain unclear.

It is known that medial prefrontal cortex (mPFC) is the key brain region mediating both social competition^8,9^and social hierarchy^10–12^. Recently, by using a food reward competition assay among two or more food-deprived animals, neuroscientists have discovered that mPFC neurons drive competitive interactions within a social group^13,14^. In mPFC, competition-related neural activity is modulated before the competition trial begins and predicts whether the trial would be a win or loss^15^. They also found that the activation of dorsalmedial prefrontal cortex (dmPFC) induces winning in social competitions by initiating and maintaining effortful behaviors, instead of enhancing aggression or strength^15^. Additionally, excitatory synaptic efficacy in dorsal mPFC bidirectionally mediates social hierarchy. Moreover, the social rank and competitive success can be predicted by the population neural dynamics in the mPFC^14^. Therefore, the excitatory neurons in mPFC may serve as an axis to encode both competitive behavior and the following dominant behaviors. However, the molecular regulatory mechanism underlying the mPFC neural ensemble remains elusive.

Homeostasis of neural activity depends on the precise regulation of numerous gene and protein expressions, which rely on diverse processes, such as transcription, translation, and alternative pre-mRNA splicing (AS)^16–18^. AS events are highly prevalent in the nervous system and play significant roles in various neuronal functions, including learning and memory, sensory information process, and social behaviors^19–22^. For example, social interaction has been shown to result in a dramatic increase in AS events in the amygdala of microbiome-deficient mice^23^. Activated orexin/hypocretin signal increases the expression level of an AS variant of α-amino-3-hydroxy-5-methylisoxazole-4-propionic acid receptor (AMPA-R) subunit in interpeduncular nucleus of starved zebrafish, and promotes zebrafish to win in social conflict^24^. Many types of RNA-binding proteins (RBPs) regulate AS, such as serine/arginine-rich proteins, heterogeneous ribonucleoprotein proteins and Celf family proteins^25^. In the case of social dominance, Celf4-mediated AS of *Syn2*pre-mRNA affects social hierarchy status through mediating AMPA-R membrane expression^26^. Beyond these examples, there is increasing evidence for the role of AS in shaping social behaviors^27–30^. However, it remains unclear whether and how AS affects competitive variance in the social group.

In this study, we first reported that during food competitive behavior, prelimbic cortex (PL) neurons could be categorized into four groups based on microelectrode recordings in freely moving animals. Most of the cells associated with competition were excitatory neurons. Using fluorescence-activated cell sorting (FACS), we analyzed AS events in excitatory neurons of the PL cortex. We discovered that the utilization of *Pkm* Exon9 and Exon10 was remodeled, resulting in a decrease in the Pkm1/2 ratio in highly competitive mice. By employing a tet-on/off system, we disrupted or rebuilt the normal Pkm1/2 ratio by controlling the expression of Pkm2 (but not Pkm1) in PL excitatory neurons. We found that the firing change of competition-related neurons occurred immediately after Pkm2 expression mediation. This alteration in firing patterns was accompanied by changes in competitive success preceding rank shifts. Furthermore, we identified a long non-coding RNA (lncRNA) named *Sera*, which was located antisense to the *Pkm* gene. *Sera* modulated the ensemble of competition-related neurons and competitive behavior by regulating the AS of *Pkm* via blocking the binding of U1 snRNP with the 5′ GU splicing site. Finally, we discovered that postsynaptic Pkm2 exerted its kinase activity by phosphorylating the Ser845 site of AMPA-R GluA1, promoting its membrane trafficking. Application of a membrane-permeable peptide to block the phosphorylation site effectively reduced the levels of membranous AMPA receptors, leading to decreased competitive success as well as social rank.

## Results

### Separate encoding of competition and rank by excitatory neurons in PL

**Fig. 1:**
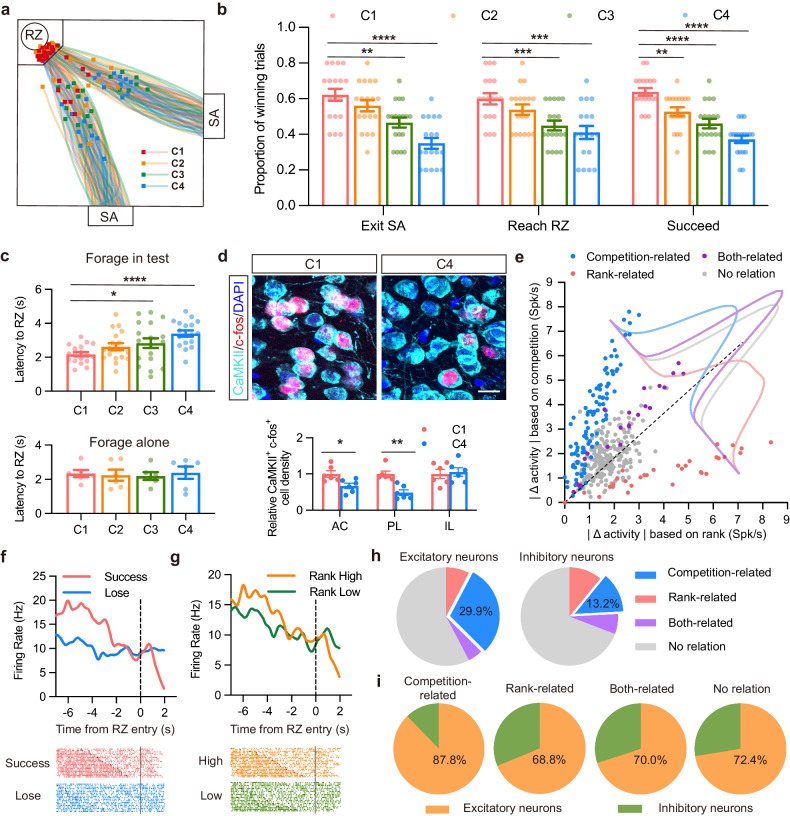
The majority of competition-related neurons in PL are excitatory neurons. **a** Mouse spatial trajectories across trials within a representative recording session. Squares indicate the instantaneous mouse positions when the first mouse entered the reward zone. RZ, reward zone; SA, starting area. **b** Winning proportions of mice exiting SA, reaching RZ and succeeding in pellet consumption. Each plot represents a session for a pair of mice. The competitive order within a social group was established (C1 to C4) based on pellet consumption proportion. *n* = 18 sessions from 6 groups. **c** Latency of mice to reach RZ. Up: each plot represents the average latency of a session for a pair of mice. *n* = 18 sessions from 6 groups. Down: each plot represents the average latency of each mouse. *n* = 6 from 6 groups. **d** Up: representative double-immunofluorescence images with antibodies against CaMKII (turquoise) and c-fos (red) in PL; down: relative CaMKII^+^c-fos^+^ cell density in anterior cingulate cortex (AC), PL and infralimbic cortex (IL) of C1 and C4 mice. *n* = 5. Bar = 30 μm. **e** Scatter plots illustrating the absolute difference in neuronal activities per neuron. Dots were color-coded based on whether they displayed significant differences in response to relative rank and competitive success. *n* = 379 from 10 mice. **f**, **g** Peri-event histogram and spike raster plots of competition-related neurons analyzed based on competitive success (**f**) and relative rank (**g**). **h**, **i** Venn diagrams depicting distribution of excitatory and inhibitory neurons based on their responses to relative rank and competitive success. *n* = 288 for excitatory neurons. *n* = 91 for inhibitory neurons. All data are presented as means ± SEM. For **b** and **c**, two-way analysis of variance (ANOVA) with Dunnett’s multiple-comparison test was used to compare with C1 groups. For **d**, paired *t*-test was used. *P < 0.05, **P < 0.01, ***P < 0.001, and ****P < 0.0001.

To measure social hierarchy within the same group, we applied the tube test, and found that the majority (76.7%) of them showed a rank fluctuation in the first few days preceding a stable linear hierarchy (Supplementary Fig. S1a‒d). It was noteworthy that the average time spent in the tube test was significantly shorter after the stable rank emerged. For those groups that showed consistent rank from the beginning, we could also find an inflection point when average time in tube tended to be stable (Supplementary Fig. S1e, f). To evaluate the competitive behaviors in group-housed mice with linear hierarchy, we carried out a territory paradigm known as the “warm spot (WS) test”, and a foraging paradigm in which four male C57/BL6 mice housed in the same cage were tested pair-wise for ten times to freely compete for food after fasting (Supplementary Fig. S1g). WS test was conducted in a cold environment to measure the occupation duration of a WS, which could be occupied by only one mouse at a time (Supplementary Fig. S1h). The competitive order in each group was strongly correlated with hierarchy rank (Supplementary Fig. S1i, j). In the training stage of food competition test, all the mice displayed the comparable latencies to reach the reward zone (RZ) when they foraged alone (Supplementary Fig. S1k, l), suggesting there is no difference in motor ability and foraging motivation among the mice. For each test trial, two paired mice were simultaneously released from the same starting area (SA), and competed to reach the RZ, which contained a single food pellet. Success in the competition was defined as the consumption of food pellet ultimately. We established a clear competitive order within the social group, denoted as C1 to C4, based on the proportion of food pellet consumption by each mouse. When mice shared the same food-winning proportion, their competitive order was determined by the average latency to reach the RZ. We noted that the competitive order remained consistent throughout various stages of the foraging race (from first exiting SA to first reaching RZ), not limited to just the final phase (Fig. 1a, b). Similar to WS test, success in food competition test was also strongly influenced by each mouse’s relative rank compared to its competitor (Supplementary Fig. S1m). Highly-ranked mice (R1) displayed comparable latencies to RZ no matter when they were foraging alone or competing with others, while low-ranked mice (R4) foraged at a slower pace when they were competing (Fig. 1c; Supplementary Fig. S1l). Together, these findings suggest that mice in a social group allocate a priority order, which is based on social hierarchy, in dividing available resources among the individuals.

It has previously been established that mPFC played a vital role in mediating both competitive behavior and social hierarchy within social group^10,14^. Therefore, we conducted c-fos staining after food competition test, and found a notably higher activation of excitatory neurons in mPFC of C1 mice, particularly in PL (Fig. 1d; Supplementary Fig. S2). To delve deeper into whether and how PL neurons encode food competition behaviors, we implanted microelectrode arrays in a mid-ranked mouse from each group to record PL neuronal activity during competitions. We successfully recorded 379 well-isolated neurons from 10 mice. By focusing on the relative rank and competitive success, we identified task-related units, including competition-related, rank-related and both-related neurons (Fig. 1e‒g; Supplementary Fig. S3). Among these neurons, 25.9% (*n*= 98) exhibited differential activity patterns based on competitive success or failure, indicating their competition-related properties. Only 8.4% (*n*= 32) showed activity variations based on whether the mouse was facing a dominant or subordinate rival. Additionally, 5.3% (*n*= 20) responded to both competition and rank. We further segregated 288 excitatory neurons and 91 inhibitory neurons based on their firing characteristics (Supplementary Fig. S4). Notably, 42.4% (*n*= 122) of excitatory neurons exhibited task-related modulation, with the majority (29.9%, *n*= 86) being associated with competition. A smaller fraction (7.6%, *n*= 22) displayed correlation with rank, and 4.9% (*n*= 14) responded to both competition and rank. Conversely, in inhibitory neurons, a smaller proportion exhibited task-related modulation. Specifically, 13.2% (*n*= 12) were linked to competition, 11.0% (*n*= 10) to rank, and 6.6% (*n*= 6) demonstrated characteristics related to both competition and rank (Fig. 1h, i). These findings suggested that activation of excitatory neurons in the PL region played a more substantial role in mediating competition behaviors compared to inhibitory neurons.

### Differential AS of Pkm in PL excitatory neurons

**Fig. 2:**
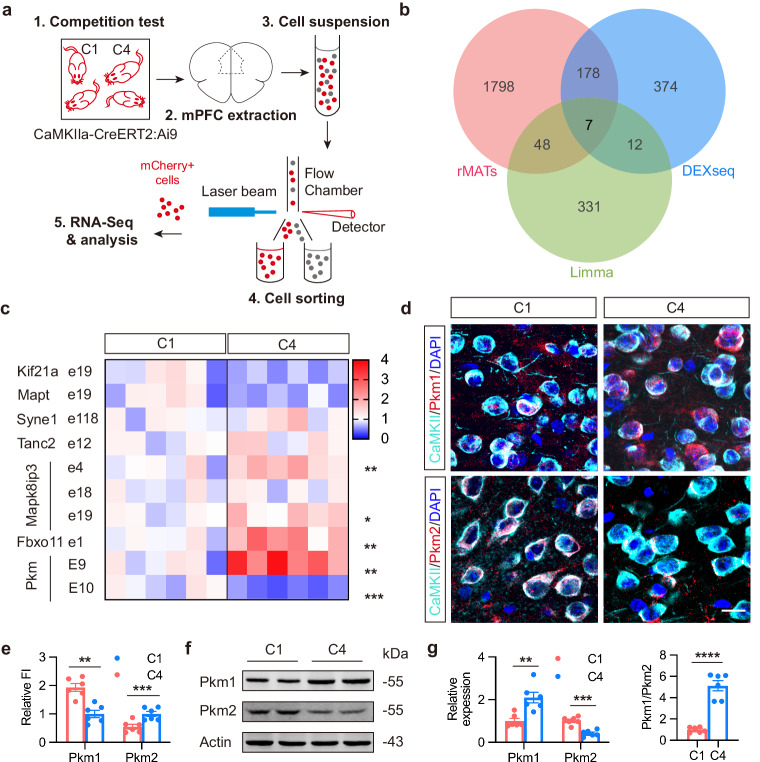
Pkm AS differences in PL excitatory neurons of highly and low competitive mice. **a** Schematic representation of separating CaMKII^+^ cells from C1 and C4 mice by FACS. **b** Veen diagram showing the number of genes with differential splicing events in mPFC of C1 and C4 mice analyzed by each tool. **c** Validation of differential splicing events in mPFC via qPCR. *n* = 6. Exon9 and Exon10 of *Pkm* were denoted to E9 and E10. Other descriptions of exon mapping, such as e18 and e19, were assessed by DEXseq. **d** Representative double-immunofluorescence images with antibodies against Pkm1 or Pkm2 (red) and CaMKII (turquoise) in PL. Bar = 30 μm. **e** Quantification for relative fluorescence intensity (FI) of Pkm1 and Pkm2 in PL. *n* = 6. **f**, **g** Representative blots (**f**) and quantification (**g**) showing differential expression of Pkm1 and Pkm2 in mPFC of C1 and C4 mice. *n* = 6. All data are presented as mean ± SEM. For **c**, **e** and **g**, paired *t*-test was used. **P < 0.01, ***P < 0.001, and ****P < 0.0001.

Mammalian neurons respond to electrical and chemical stimulation with multiple AS events, which are crucial for accomplishing the complex functions, including the regulation of critical proteins essential for synapse formation and maintenance^31^. To analyze differences of AS signatures in excitatory neurons of PL during competitive behaviors, we isolated the excitatory neurons as labeled by mCherry from the *CaMKIIa-CreERT2:Ai9*mice with high and low competitiveness and sent for RNA-sequencing (Fig. 2a). After alignment, we employed three distinct tools to analyze the splicing events: exon-based DEXSeq, limma, and event-based rMATs. We revealed ten differentially regulated exons within seven genes (Fig. 2b; Supplementary Fig. S5). Among them, the AS of *Pkm* mRNA is of particular interest, because (1) In low competitive mice, *Pkm* Exon9 usage was upregulated while Exon10 usage was downregulated (Fig. 2c). These two exons displayed the most significant usage difference among the ten exons analyzed; (2) Compared to other genes, only the splicing in *Pkm* mRNA results in different functional proteins; (3) *Pkm* encodes the Pkm1 and Pkm2 isoforms through AS of mutually exclusive Exon9 and Exon10, and such alteration of splicing in *Pkm* gene leads to the differential expression of Pkm1 and Pkm2, which play distinct roles in metabolic reprogramming^32^, a critical event in synapse. Indeed, in low competitive mice, the expression of Pkm1 protein was upregulated, while Pkm2 was downregulated. Increased Pkm1/2 ratio in low competitive mice was consistent with the observed AS pattern of *Pkm*(Fig. 2d‒g). Immunofluorescence staining further validated that differential exon usage in *Pkm* was predominantly confined to excitatory neurons within the PL region of the brain, in both highly and low competitive mice. This pattern closely mirrored the pattern of neuronal activation.

### Pkm2, but not Pkm1, shapes social competition and hierarchy

**Fig. 3:**
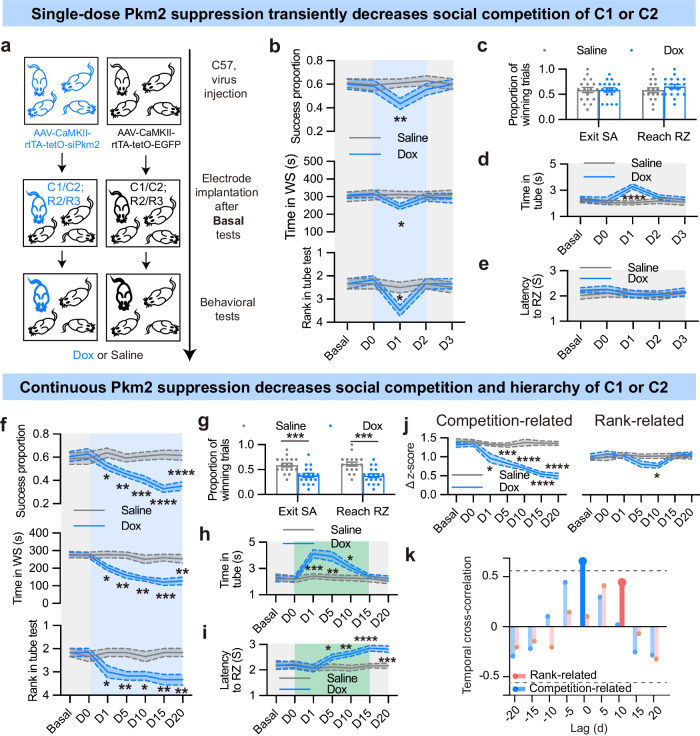
Continuous Pkm2 suppression in PL decreases social competition and social hierarchy. **a** Schematic illustration of the experiment. **b** Behavioral recordings of highly competitive C57 mice injected with AAV-CaMKII-rtTA-tetO-siPkm2-EGFP and applied with single-dose Dox. Top: winning proportions of pellet consumption in food competition test, *n* = 18 sessions from 6 groups; middle: occupation time of WS in WS test, *n* = 6 from 6 groups; bottom: rank in tube test, *n* = 6 from 6 groups. **c** Winning proportions of mice exiting SA and reaching RZ on day 3. *n* = 18 sessions from 6 groups. **d** Average time in the tube test. *n* = 6. **e** Latency of mice to reach RZ. *n* = 18 sessions from 6 groups. **f** Behavioral recordings of highly competitive C57 mice injected with AAV-CaMKII-rtTA-tetO-siPkm2-EGFP and applied with Dox for 21 days. Top: winning proportions of pellet consumption in food competition test, *n* = 18 sessions from 6 groups; middle: occupation time of WS in WS test, *n* = 6 from 6 groups; bottom: rank in tube test, *n* = 6 from 6 groups. **g** Winning proportions of mice exiting SA and reaching RZ on day 20. *n* = 18 sessions from 6 groups. **h** Average time in the tube test. *n* = 6. **i** Latency of mice to reach RZ. *n* = 18 sessions from 6 groups. **j** Neuronal recordings of highly competitive mice. Left: Δz-score of competition-related neuron firing rate; right: Δz-score of rank-related neuron firing rate. *n* = 6 from 6 groups. **k** Temporal cross-correlation between changes in food competition and neuronal encoding. Deep blue line indicates that time lag was significant. All data are presented as means ± SEM. For **b**, **d**, **e**, **f** and **h**‒**j**, two-way ANOVA with Bonferroni analysis was used. For **c** and **g**, unpaired *t*-test was used. *P < 0.05, **P < 0.01, ***P < 0.001, and ****P < 0.0001.

To investigate the causal relationship between differentially expressed Pkm isoforms in excitatory neurons of the PL region and their impact on neuronal encoding and mouse competitiveness, we employed a Tet-on system to manipulate the expression of Pkm2 in these neurons. Specifically, we injected the AAV-CaMKII-rtTA-tetO-siPkm2 virus into the PL region of highly competitive C57 mice to regulate the expression of Pkm2 (Fig. 3a). Immunofluorescence staining showed that Pkm2 knockdown decreased Pkm2 expression without affecting Pkm1 (Supplementary Fig. S6). Behavioral tests were conducted to determine the basal hierarchy and competitive order of mice 3 weeks after virus injection. Subsequently, for each cage, a mid-ranked (R2 or R3) mouse with higher competitiveness in food competition (C1 or C2) were implanted with electrodes and then administrated with either doxycycline (Dox) or saline (Fig. 3a). Single-dose Dox application on day 0 decreased competition success and rank in tube test on the next day, while mice returned to the original level on day 2 and 3 (Fig. 3b). Winning trial proportion of mice applied with saline or Dox in exiting SA and reaching RZ showed no difference on day 3 (Fig. 3c). Neither the latency to reach RZ nor time spent in tube showed a long-term change (Fig. 3d, e), indicating that transient Pkm2 mediation only changed social competition, but not hierarchy. In long-term experiment, Dox was applied for 21 continuous days. Behavioral tests and neuronal recordings were carried out on day 0, day 1, day 5, day 10, day 15 and day 20. We found that competitive success of Dox-treated mice (with lower Pkm2 expression) progressively declined over time. Compared with saline-treated mice, Dox-treated mice obtained fewer food pellets and spent less time on the WS from day 1 on (Fig. 3f, g), suggesting a reduction of competitive ability. Rank of Dox-treated mice assessed by tube test also changed from day 1 on (Fig. 3f). However, changed rank in tube test on the initial point did not equal to a synchronous alteration of social hierarchy. Established social hierarchy is considered to reduce long-term conflict between conspecifics. As we described above, social hierarchy was considered as the stable rank over time, accompanied with a relative shorter time spent in the tube test, as well as the success proportion and differential latency to reach RZ in food competition test. After Dox application, average time spent in tube significantly increased from day 1 to 10, but returned to a stable low level on days 15 and 20 (Fig. 3h). Similarly, latency of the Dox-treated mice to reach RZ in food competition test gradually increased from day 5 on, and maintained at a longer level after day 15 (Fig. 3i). These results suggested that original stable social hierarchy underwent great challenge and re-established gradually from day 1 to 15. And continuous competition alternation eventually resulted in social hierarchy reestablishment.

Furthermore, Dox-treated mice displayed a significant decrease in the firing rate of competition-related neuron over time, while mice treated with saline did not. Additionally, there was a decrease in the firing rate of rank-related neurons in Dox-treated mice on day 10 (Fig. 3j). To assess the relationship between behavior and neural activity changes, we aligned and compared behavioral and neuronal data over a 21-day period in Dox-treated mice to describe their statistical dependency. Cross-correlation analysis revealed a positive and significant relationship between competition-related neuron activity and competition without lag, suggesting that changes in competition closely followed changes in competition-related neuronal encoding. Although not statistically significant, the peak time lag between changes in rank-related and competition-related neuron activity was 10 days (Fig. 3k).

**Fig. 4:**
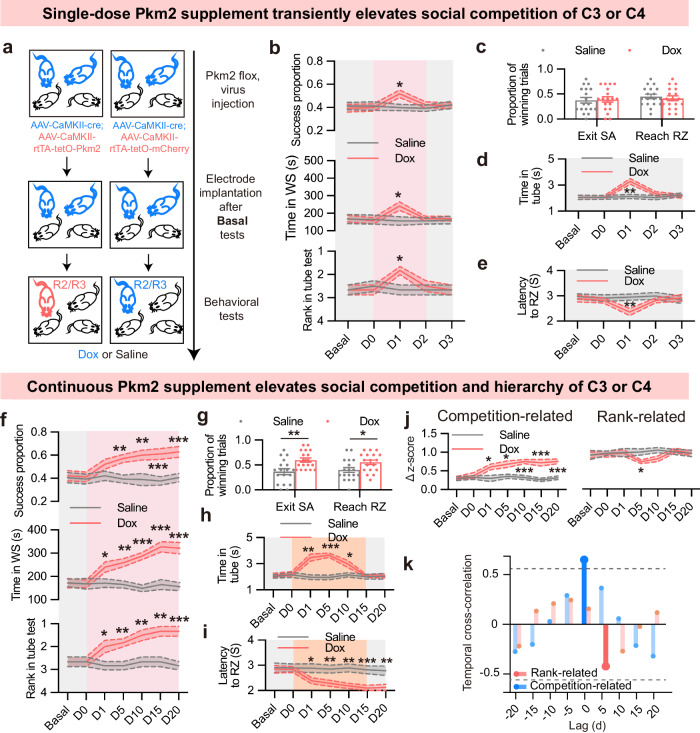
Continuous Pkm2 supplement in PL elevates social competition and social hierarchy. **a** Schematic illustration of experiment. **b** Behavioral recordings of Pkm2-flox mice injected with AAV-CaMKII-Cre-EGFP and AAV-CaMKII-rtTA-tetO-Pkm2-mCherry, and applied with single-dose Dox. Top: winning proportions of pellet consumption in food competition test, *n* = 18 sessions from 6 groups; middle: occupation time of WS in WS test, *n* = 6 from 6 groups; bottom: rank in tube test, *n* = 6 from 6 groups. **c** Winning proportions of mice exiting SA and reaching RZ on day 3. *n* = 18 sessions from 6 groups. **d** Average time in the tube test. *n* = 6. **e** Latency of mice to reach RZ. *n* = 18 sessions from 6 groups. **f** Behavioral recordings of Pkm2-flox mice injected with AAV-CaMKII-Cre-EGFP and AAV-CaMKII-rtTA-tetO-Pkm2-mCherry, and applied with Dox for 21 days. Top: winning proportions of pellet consumption in food competition test, *n* = 18 sessions from 6 groups; middle: occupation time of WS in WS test, *n* = 6 from 6 groups; bottom: rank in tube test, *n* = 6 from 6 groups. **g** Winning proportions of mice exiting SA and reaching RZ on day 20. *n* = 18 sessions from 6 groups. **h** Average time in the tube test. *n* = 6. **i** Latency of mice to reach RZ. *n* = 18 sessions from 6 groups. **j** Neuronal recordings. Left: Δ z-score of competition-related neuron firing rate; right: Δ z-score of rank-related neuron firing rate. *n* = 6 from 6 groups. **k** Temporal cross-correlation between changes in food competition and neuronal encoding. Deep blue line indicates that time lag was significant. All data are presented as means ± SEM. For **b**, **d**, **e**, **f** and **h**‒**j**, two-way ANOVA with Bonferroni analysis was used. For **c**, **g**, unpaired *t*-test was used. *P < 0.05, **P < 0.01, ***P < 0.001, and ****P < 0.0001.

Then, we generated the Pkm2 knockdown (Pkm2 KD) mice by injecting the AAV-CaMKII-Cre-EGFP virus into the PL excitatory neurons of random two from four co-housed Pkm2-flox mice. Concurrently, we injected AAV-CaMKII-rtTA-tetO-Pkm2 to Pkm2 KD mice to timely restore the expression of Pkm2 without affecting Pkm1 (Fig. 4a; Supplementary Fig. S7a‒d). In baseline tests, Pkm2 KD mice showed a tendency towards lower competitive ability and lower dominance ranking in their hierarchies (Supplementary Fig. S7e‒g). Single-dose Dox application on day 0 transiently increased competition success and rank in tube test on the next day, without alteration of social hierarchy (Fig. 4b‒e). Following long-term Dox treatment, the Pkm2 KD mouse with a middle rank in each group gradually regained high competitiveness and eventually restored social dominance (Fig. 4f‒i). Cross-correlation analysis also confirmed the positive and significant relationship between competition-related neuron activity and competition (Fig. 4j, k). Similarly, Pkm2 supplement in low competitive wild-type (WT) mice also increased their competitiveness and social rank (Supplementary Fig. S8). Furthermore, Dox treatment alone did not alter competition behavior in highly competitive mice (Supplementary Fig. S9a‒f) or Pkm2 KD mice (Supplementary Fig. S9g‒l).

Given the apparent downregulation of Pkm1 in the highly competitive mice, we therefore injected AAV-CaMKII-rtTA-tetO-Pkm1 into the PL region of C1 or C2 mice to overexpress Pkm1 without affecting Pkm2 (Supplementary Fig. S10a‒e). After Dox treatment, these mice were subjected to the competitive tests and no significant difference in the competitive behaviors or rank shift was detected (Supplementary Fig. S10f). Knockdown of Pkm1 in PL of C3 or C4 mice also did not alter the competitiveness and social rank (Supplementary Fig. S11). These findings strongly suggested that Pkm2, but not Pkm1, played a role in the competition-related neuronal encoding capacity and the competition success.

### LncRNA *Sera* mediates *Pkm* AS

**Fig. 5:**
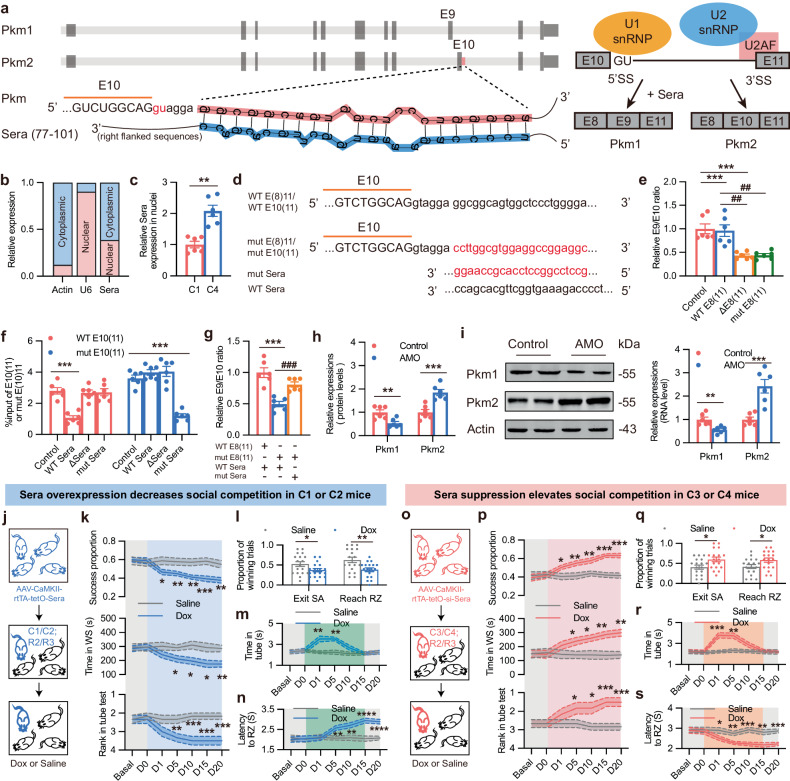
*Sera* mediates competition and hierarchy through *Pkm* AS. **a** Hypothetical mechanism of *Sera* mediating *Pkm* AS. Left: predicted binding sites between *Pkm* pre-mRNA and *Sera* via RNAcofold; right: a model for *Sera* mediating *Pkm* AS. **b** Cytoplasmic/nuclear ratio of *Sera* was detected by subcellular fractionation and qPCR. *n* = 6. **c** Nuclear *Sera* expression in mPFC of C1 and C4 mice via qPCR. *n* = 6. **d** Mutation of *Sera* and E8(11) or E10(11). **e** Exon usage was detected by qPCR 48 h after plasmids expressing WT E8(11), ΔE8(11) and mut E8(11) were separately transfected into N2a cells. *n* = 6. **f** qPCR analysis of WT E10(11) or mut E10(11) binding to U1 snRNP. Plasmids expressing WT E10(11) or mut E10(11) with WT *Sera*, Δ*Sera* or mut *Sera* were separately transfected into N2a cells, and RIP was carried out after 48 h. *n* = 6. **g** Exon usage was detected by qPCR 48 h after plasmids expressing WT E8(11) or mut E8(11), together with WT *Sera* or mut *Sera* were transfected into N2a cells. *n* = 6. **h** RNA level of *Pkm1* and *Pkm2* was detected by qPCR, 48 h after AMO targeting the predicted binding sequence of *Pkm* pre-mRNA was transfected to N2a cells. *n* = 6. **i** Representative blots (left) and quantification (right) of Pkm1 and Pkm2 protein after AMO was transfected to N2a cells. *n* = 6. **j** Schematic illustration of experiment. **k** Behavioral recordings of highly competitive C57 mice injected with AAV-CaMKII-rtTA-tetO-*Sera*-mCherry. Top: winning proportions of pellet consumption in food competition test, *n* = 18 sessions from 6 groups; middle: occupation time of WS in WS test, *n* = 6 from 6 groups; bottom: rank in tube test, *n* = 6 from 6 groups. **l** Winning proportions of mice exiting SA and reaching RZ on day 20. *n* = 18 sessions from 6 groups. **m** Average time in the tube test. *n* = 6. **n** Latency of mice to reach RZ. *n* = 18 sessions from 6 groups. **o** Schematic illustration of experiment. **p** Behavioral recordings of low competitive C57 mice injected with AAV-CaMKII-rtTA-tetO-si-*Sera*-mCherry. Top: winning proportions of pellet consumption in food competition test, *n* = 18 sessions from 6 groups; middle: occupation time of WS in WS test, *n* = 6 from 6 groups; bottom: rank in tube test, *n* = 6 from 6 groups. **q** Winning proportions of mice exiting SA and reaching RZ on day 20. *n* = 18 sessions from 6 groups. **r** Average time in the tube test. *n* = 6. **s** Latency of mice to reach RZ. *n* = 18 sessions from 6 groups. All data are presented as mean ± SEM. For **e,** **f** and **g**, two-way ANOVA with Dunnett’s multiple-comparison test was used. For **c**, paired *t*-test was used. For **h,** **i,** **l** and **q**, unpaired *t*-test was used. For **k,** **m,** **n,** **p,** **r** and **s**, two-way ANOVA with Bonferroni analysis was used. *P < 0.05, **P < 0.01, ***P < 0.001, and ****P < 0.0001.

Previous studies suggest that lncRNA can regulate AS of pre-mRNA, either directly or indirectly^33,34^. Therefore, we explored the potential upstream lncRNAs that could regulate the *Pkm* AS. Combined with the RNA sequencing data, we identified 6 lncRNA with cis-regulation potential to *Pkm* mRNA. qPCR validation further confirmed that *AK016725*, *AK132182* and *AK018848* were increased in mPFC of low competitive mice (Supplementary Fig. S12a). However, only *AK018848* was mainly distributed in the excitatory neurons (Supplementary Fig. S12b). In vivo FISH experiments with Pkm1 or Pkm2 co-staining, further confirmed downregulation of *AK018848* in PL of highly competitive mice and its correlations with Pkm1/2 expressions (Supplementary Fig. S12c‒g). In cultured neurons, *AK018848* was verified to increase Pkm1/2 ratio through positively regulating Pkm1 and negatively regulating Pkm2 (Supplementary Fig. S12h‒k). Like other lncRNAs, *AK018848* scored very low in both algorithms when compared to known coding transcripts. A further biochemical study confirmed that *AK018848* had no coding capacity (Supplementary Fig. S13). Considering that *AK018848* was the potential splicing-related lncRNA that located in the antisense of *Pkm*, we then renamed it as *Sera*. To investigate the potential mechanism by which *Sera* regulates *Pkm* AS, we analyzed the possible binding sites between *Sera* and *Pkm* pre-RNA. We found that a 25-nt seed sequence of *Sera* (located between site 77 and 101) could bind with the intron 10 after Exon 10 of *Pkm* pre-RNA (Fig. 5a). This binding site was situated near the 5′ GU splicing site, which was recognized by U1 snRNP and crucial for the AS of *Pkm*. Based on these findings, we hypothesized that *Sera* binding to *Pkm* pre-RNA may interfere with U1 snRNP recognition of the 5′ GU splicing site, thus promoting Exon 9 retention and Exon 10 exclusion.

To test this hypothesis, we first detected subcellular location of *Sera* in mPFC via qPCR and found that 38.6% was located in the nuclei (Fig. 5b). qPCR validation further confirmed that nuclear *Sera* was increased in mPFC of low competitive mice (Fig. 5c). Next, we constructed plasmids expressing *Pkm* WT E8(11) (full-length DNA with introns from Exon 8 to Exon 11, Chr9: 59579204-59585508), ΔE8(11) [E8(11) without the predicted binding site] and mut E8(11) [E8(11) with mutation of the predicted binding site] (Fig. 5d). We observed that the E9/E10 retaining ratio decreased in both ΔE8(11)-transfected and mut E8(11)-transfected N2a cells (Fig. 5e), suggesting that the predicted binding site in *Pkm* pre-mRNA was critical for AS. We also constructed plasmids expressing *Pkm* WT E10(11) (full-length RNA with introns from Exon 10 to Exon 11, Chr9: 59582312-59583017), mut E10(11), full-length WT *Sera*, Δ*Sera* (*Sera* without predicted seed sequence) and mut *Sera* (*Sera* with complimentary mutation to bind the mutated intron 10 binding sites) (Fig. 5d), and transfected these plasmids into the N2a cells. Then, RNA immunoprecipitation (RIP) assay with an antibody against U1 snRNP was performed. We found that fewer WT E10(11) RNAs bound to U1 snRNP in WT *Sera*-transfected cells than control, and the decrease of binding was rescued in Δ*Sera* and mut *Sera* groups (Fig. 5f). Binding efficiency of mut E10(11) showed no difference among the control, WT *Sera* and Δ*Sera* groups, while a significant decrease was observed in the mut *Sera*-transfected group (Fig. 5f), suggesting that binding interaction between *Sera* and *Pkm* pre-RNA impeded U1 snRNP recognizing *Pkm* intron 10. To test whether complimentary mutation of *Sera* rescued the decreased splicing of mut E8(11), we detected E9/E10 retaining ratio after transfection and found that reduced E9/E10 ratio in mut E8(11) group co-transfected with WT *Sera* was partially rescued when mut E8(11) was co-transfected with mut *Sera* (Fig. 5g). However, 5′ splicing site of intron 10 was not directly bound with *Sera*. Therefore, we hypothesized that right flanked sequences (after site 101) of *Sera* seed sequence might suppress binding of U1 snRNP with 5′-GU. We designed an antisense 2′-O-methoxyethyl modified oligonucleotide (AMO) to target the predicted binding sequence of intron and found that E10 retention was promoted after AMO transfection, resulting in an increase of Pkm2 and a decrease of Pkm1 in cells treated with AMO (Fig. 5h, i). These results suggest that competitive binding of AMO with intron 10 inhibits the suppression of U1 snRNP binding with 5′-GU mediated by *Sera*. Together, these data suggest that *Sera* could regulate the splicing of *Pkm* pre-mRNA by specifically binding with the specific sequence after Exon10.

To determine whether *Sera* played a role in regulating competitive behavior and social hierarchy, we bidirectionally manipulated *Sera* expression in PL excitatory neurons. We injected the AAV-CaMKII-rtTA-tetO-Sera-mCherry virus into the PL region of highly competitive C57 mice (Fig. 5j). FISH staining and nuclear qPCR confirmed nuclear overexpression of *Sera* (Supplementary Fig. S14a‒c). *Sera* overexpression successfully increased Pkm1 expression and decreased Pkm2 expression in vivo (Supplementary Fig. S14d‒h). Following long-term Dox treatment, competitive success of Dox-treated mice progressively declined over time. (Fig. 5k, l). Average time of the Dox-treated mice spent in tube returned to a stable low level on day 15 and 20 (Fig. 5m), while latency to reach RZ gradually increased from day 5 on (Fig. 5n), suggesting that competition change was anterior to rank change. Next, we injected the AAV-CaMKII-rtTA-tetO-si-Sera-mCherry virus into the PL region of low competitive C57 mice. *Sera* knockdown in low competitive mice decreased Pkm1 expression and increased Pkm2 expression (Supplementary Fig. S14i‒p) and progressively improved their competitiveness and rank (Fig. 5o‒s). As expected, the control virus injection and Dox treatment did not affect the social competition and rank in low competitive mice (Supplementary Fig. S15). In summary, we found that *Sera* not only mediated *Pkm* AS in vivo, but also influenced competitive success and hierarchy sequentially.

### Pkm2 mediates competition behaviors via GluA1 phosphorylation

**Fig. 6:**
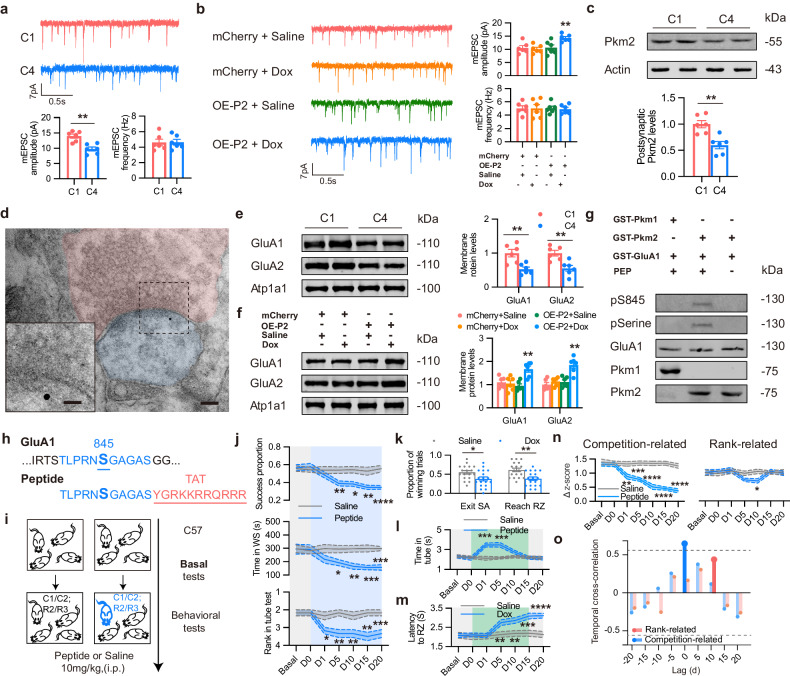
Pkm2 promotes GluA1 membrane trafficking through phosphorylation. **a** Representative traces of mEPSCs from PL neurons of C1 and C4 mice. Down: the mean mEPSC amplitude and frequency were analyzed. *n* = 30 neurons from 6 mice for each group. **b** Representative traces of mEPSCs from PL neurons of Pkm2 KD mice overexpressed with tet-on Pkm2 or mCherry, with or without Dox application. Right: the mean mEPSC amplitude and frequency were analyzed. *n* = 30 neurons from 6 mice for each group. **c** Postsynaptic fractions were extracted from mPFC C1 and C4 mice, and then subjected to immunoblot. *n* = 6. **d** Representative immunoelectron microscope image for mouse mPFC staining with anti-Pkm2. Bar = 100 nm or 30 nm. **e** Representative blots (left) and quantification (right) for membrane GluA1 and GluA2 of mPFC from C1 and C4 mice. *n* = 6. **f** Representative blots (left) and quantification (right) for membrane GluA1 and GluA2 of mPFC that was overexpressed with Pkm2. *n* = 6. **g** Phosphorylation of GST-GluA1 by Pkm1 or Pkm2 with or without PEP as phosphate donor was revealed by immunoblot assays using antibody against S845 phosphorylated GluA1 and phosphorylated serine. *n* = 3. **h** Design of peptide blocking phosphorylation of GluA1 S845. **i** Schematic illustration of experiment. **j** Behavioral recordings of C1 and C2 mice applied with peptide or saline. Top: winning proportions of pellet consumption in food competition test, *n* = 18 sessions from 6 groups; middle: occupation time of WS in WS test, *n* = 6 from 6 groups; bottom: rank in tube test, *n* = 6 from 6 groups. **k** Winning proportions of mice exiting SA and reaching RZ on day 20. *n* = 18 sessions from 6 groups. **l** Average time in the tube test. *n* = 6. **m** Latency of mice to reach RZ. *n* = 18 sessions from 6 groups. **n** Neuronal recordings. Left: Δ z-score of competition-related neuron firing rate; right: Δ z-score of rank-related neuron firing rate. *n* = 6 from 6 groups. **o** Temporal cross-correlation between changes in food competition and neuronal encoding. Deep blue line indicates that time lag was significant. All data are presented as means ± SEM. For **a**, **c**, **e** and **k**, *t*-test was used. For **b** and **f**, two-way ANOVA with Dunnett’s multiple-comparison test was used. For **j**, **l**, **n**), two-way ANOVA with Bonferroni analysis was used. *P < 0.05, **P < 0.01, ***P < 0.001, and ****P < 0.0001.

To understand the downstream mechanisms by which Pkm2 mediates competition, we first examined key metabolites in glycolysis but found no significant differences between highly and low competitive mice (Supplementary Fig. S16). Given the critical role of synaptic plasticity in neural coding and information processing^35^, especially AMPA-R-dependent synaptic strength in social competition^24^, we proceeded to compare the miniature excitatory postsynaptic currents (mEPSCs) in the PL excitatory neurons between highly and low competitive mice. We found a relative decrease in mEPSC amplitude in low competitive (C4) mice (Fig. 6a). Moreover, increased amplitude but comparable frequency of mEPSCs was found after Pkm2 supplementation in the PL of Pkm2 KD mice and C57 low competitive mice (Fig. 6b; Supplementary Fig. S17a, b), consistent with the results for mice that show higher competition. Knockdown of Pkm2 in highly competitive mice also decreased mEPSCs amplitude without changing frequency (Supplementary Fig. S17c, d). These findings suggest a potential role of Pkm2 in the postsynaptic function. Next, we isolated various synaptic fractions and found that postsynaptic Pkm2 was upregulated in C1 (Fig. 6c). Localization of Pkm2 in post-synapse was confirmed by immunoelectron microscopy, with gold particle labeled Pkm2 observed in postsynaptic density (PSD) (Fig. 6d; Supplementary Fig. S18). We then analyzed the proteins that directly interacted with Pkm2 by employing mass spectrum after immunoprecipitation with Pkm2 antibody in the PSD extracts from mPFC. Notably, we identified three subunits of AMPA-Rs (GluA1, GluA2, GluA3) and two subunits of N-methyl-D-aspartate receptors (NMDA-Rs) (Nr1, Nr2b) among 1295 identified proteins (Supplementary Fig. S19). We then examined the membranous expression of these receptors and found that GluA1 and GluA2 were reduced in the membrane fraction in the low competitive mice (Fig. 6e), as well as in the mice with Pkm2 knockdown (Supplementary Fig. S20a, b). And Pkm2 supplement rescued decreased membranous expression of GluA1 and GluA2 (Fig. 6f). By contrast, no change was found in membranous expression of the other 3 subunits and the total expression of all these 5 receptors (Supplementary Fig. S20a‒f). In line with it, *Sera* downregulation, which led to an increase in Pkm2, promoted GluA1 and GluA2 membrane trafficking, while *Sera* upregulation repressed their membrane trafficking (Supplementary Fig. S20g‒j). However, neither overexpression nor silencing of Pkm1 affected the AMPA-Rs trafficking (Supplementary Fig. S20k‒n). Emerging evidence has shown that Pkm2 also possesses protein kinase activity, besides its role in glycolysis regulation^36–38^. It is known that phosphorylation of GluA1 S845 is required for GluA1 targeting to the cell surface and its stabilization. Therefore, we tested whether Pkm2 could directly phosphorylate GluA1. An in vitro phosphorylation assay suggested that Pkm2, but not Pkm1, directly phosphorylated GluA1 at S845 in the presence of Phosphoenolpyruvate (PEP) as a phosphate donor (Fig. 6g). Mass-spectrometry analysis also confirmed that Pkm2 phosphorylated GluA1 at S845 (Supplementary Fig. S21). These results indicated that Pkm2, not Pkm1, could act as a protein kinase to phosphorylate GluA1. In addition, we designed a membrane-permeable blocking peptide to reduce the membranous AMPA-R levels (Fig. 6h; Supplementary Fig. S22). Highly competitive mice injected with peptide experienced a rapid reduction of competitiveness (Fig. 6j, k). Average time of peptide-treated mice spent in tube returned to a stable low level on day 15 and 20 (Fig. 6l), whereas latency to reach RZ gradually increased from day 5 on (Fig. 6m), suggesting that competition change was anterior to rank change. In addition, the cross-correlation analysis proved that competition changes timely followed competition-related neuronal encoding changes (Fig. 6n,o).

## Discussion

In social animals with established hierarchies, higher-ranked males have preferential access to resources, including food, territory, and reproductive opportunities^39,40^. In species with small group sizes and stable group membership, the social rank is relatively stable and minimizes intense fights within the group owing to unprovoked submissive interactions of subordinates^41,42^. When food resources are limited in quantity or quality, dominant animals have increased access to highest quantity or most profitable foods. A filed experiment on golden snub-nosed monkeys showed that dominant monkeys spent less time feeding and consumed provisioned foods at a higher rate in winter when natural foods were limited. And the reduction in feeding time allowed dominant monkeys to spend more time engaged in affiliative behaviors to promote social cohesion^43^. In the home cage of non-food deprived rats, the introduction of a new feeder with limited palatable pellets triggered asymmetric social interactions and social rank can be reliably predicted based on food consumption^44^. Consistent with these findings, we found that competition success was strongly influenced by social hierarchy within a social group of fasting mice. Dominant mice had a higher proportion of food pellet consumption, and competitive order remained consistent throughout various stages of the foraging race. Notably, no difference in latency of dominant R1 mice was found when they foraged alone or competed with subordinates, whereas R4 mice showed apparent submission in competition through reaching RZ at a lower pace. These results indicate that social status within a group was the best predictor of competition success.

Once a social hierarchy is established within a group, it remains stable over a long period, allocating a strict priority order to the individuals^45,46^. However, during the formation of social hierarchy, individuals with little knowledge of each other display varying abilities in winning agonistic interactions^2^. The outcome of aggressive contests exerts significant effects on subsequent contests, with previous winners or losers being more likely to win or lose, which is known as winner-loser effects^47–49^. Previous evidence supporting winner-loser effects extends across a wide range of animals, such as mammals, invertebrates, fish, birds and so on^50–54^. Recently, a population observation over 15,000 dyadic interactions of wild savannah baboons also suggest that winner-loser effects influenced male hierarchy in wild populations^55^. An important aspect of winner-loser effects is that the influence of a few contests is generally short lived, while continuous winning or losing experiences have long-lasting consequences and eventually lead to alterations in social status^56,57^. In subdominant mice, optogenetic activation of dmPFC results in instantaneous winning in tube test and WS competitions. Mice that received fewer than five photostimulated wins failed to maintained elevated social status. However, mice that received more than six photostimulated wins continued to win in the following days even without photostimulation. This enduring effect was due to the long-term synaptic strengthening of mediodorsal thalamus-dmPFC circuit following repeated victories^15^. Conversely, repeated forced loss in tube test activated burst firing in lateral habenula, inhibiting the neural activity in mPFC and causing a stable decrease in social rank of dominant mice^10^.

In this study, we found an immediate change in the firing rate of competition-related neurons in PL following the modulation of Pkm2 expression in excitatory neurons by using a tet-on/off system. Mice received a short-term modulation experienced a transient competitiveness alteration without hierarchy change, while a long-term competition change disturbed social hierarchy. The latency to reach RZ immediately shortened on day 1 after Pkm2 was overexpressed or *Sera* was knocked down in low competitive mice, and the average time spent in the tube test extended simultaneously from day 1 on, suggesting that increased competitiveness of low competitive mice motivated their attempt on seeking for a higher rank in the social group. However, when competitiveness was repressed in highly competitive mice, they did not make a concession to reach RZ at a slower pace on day 1, even though their final winning proportion in competition decreased. Instead, the latency extended from day 5 on, indicating that highly competitive mice might not show decreased competitiveness on their own initiative but through defeats in competition. In long-term modulation, cross-correlation analysis revealed a positive and significant relationship between the changes in activity of competition-related neurons and alterations in competition success, with no time lag, indicating a strong association between PL competition-related neurons and competition success. Reestablished social status of mice assessed by tube test rank and submissive interaction of subordinates remained relatively stable 2 weeks after competition alteration, providing further evidence of winner-loser effect.

Although not statistically significant, the peak time lag between rank-related neuron activity and competition success was delayed compared with lag between competition-related neuron activity and competition success. Notably, rank-related neurons in PL were defined as neurons that changed their activity according to whether the mouse was facing to a more dominant or subordinate one in the food competition test. The fluctuation of rank-related neuron activity possibly reflected rank recognition alternation rather than direct changes in social hierarchy. Taken together, our findings suggest that Pkm2 expression in PL excitatory neurons of mice mediates competition success and further influences social hierarchy.

Neuronal excitability and synaptic efficacy rely on an array of molecular recognition events involving ion channels and synaptic proteins^58^. In addition to altered gene expression levels of these proteins, AS serves as another important mechanism to modulate transcriptional signature and synaptic plasticity. AS, which enriches protein diversity and phenotypic traits through generating multiple transcripts from a single gene, is a highly dynamic process to allow rapid responses to stimuli^17,59^. For instance, neurexins, which are highly homologous presynaptic cell-adhesion molecules, undergo extensive AS^60,61^. AS of *neurexin-1* (*Nrxn1*) and *neurexin-3* (*Nrxn3*) at splice site 4 (SS4) involves inclusion or exclusion of a 90 bp exon, resulting in SS4^+^ or SS4^‒^ isoforms. Constitutive inclusion of *Nrxn3* SS4 reduces postsynaptic AMPA-R levels and enhances postsynaptic AMPA-R endocytosis, without affecting NMDA-R properties. By contrast, constitutive inclusion of *Nrxn1* SS4 selectively enhances responses of NMDA-R rather than AMPA-R^62–64^. In addition to presynaptic constituents, alternative spliced variants of postsynaptic proteins also participate in synaptic responses. It is known that all GluA subunits of AMPA-R are known to undergo AS, generating flip or flop variants with mutually exclusive exons to influence AMPA-R kinetics^65–67^. In a study involving male zebrafish subjected to a 6-day’s starvation, it was observed that input from orexin/hypocretin neurons could enhance the expression of AS variant GluA3b-flip. This variant exhibited a longer desensitization period, thereby prolonging AMPA-R activity in interpeduncular nucleus, ultimately increasing the winning rate of zebrafish in social conflict^24^. In this study, we demonstrated that the deregulation of *Pkm* AS by lncRNA *Sera* modulates PL neuron activity and social competition. We found that Pkm1/2 ratio was decreased in PL excitatory neurons of highly competitive mice. The enhancement of Pkm2 expression directly results in the upregulation of firing patterns of competition-related neurons, which are associated with increases in competitive success.

Emerging evidence has demonstrated that lncRNAs play a significant role in social behaviors by regulating the AS of specific targets. Non-coding RNAs can contribute to AS regulation in multiple ways, such as directly interacting with pre-mRNAs or the transcribed genomic locus, modifying chromatin accessibility by recruiting or impeding chromatin-modifying complexes, and indirectly regulating the activity of splicing factors, a category of RBPs that recognize regulatory elements within exons and introns^68–70^. For example, specific isoforms of lncRNA *Meg3*, particularly *Meg3*-ex10, were negatively affected by social fear conditioning, but were restored after extinction. In vivo knockdown of *Meg3*-ex10 before social fear acquisition delayed social fear extinction^71^. Moreover, our previous study reported that lncRNA *AtLAS* affects social dominance via alternative polyadenylation of *synapsin 2* (*Syn2*). In the mPFC of subordinate mice, overloaded AtLAS bound with *Syn2* pre-mRNA and inhibited Celf4-mediated AS to increase expression of Syn2b, which in turn restrained membrane insertion of AMPA-R^26^. Here, we identified that lncRNA *Sera* is specifically upregulated in the excitatory neurons of mPFC in the lower competitive mice. Interestingly, we screened out that *Sera* could bind with *Pkm* pre-RNA at the intron after Exon10, which is adjacent to the 5′ GU splicing site that recognized by U1 snRNP. This combination effectively disrupted the U1 snRNP-dependent AS of *PKM* pre-RNA and led to an increase in the Pkm1/2 ratio. In highly competitive mice, decreased *Sera* levels result in an increase in Pkm2 expression. Additionally, specific overexpression or knockdown of *Sera* in excitatory neurons of the prelimbic cortex (PL) bidirectionally regulates competitive behaviors through *Pkm* AS.

Pkm1/2 is a key molecule of glycolytic system, resulting from mutually exclusive AS of the *Pkm* pre-mRNA. Pkm1/2 plays a crucial role in the final and rate-limiting step of glycolysis, converting phosphoenolpyruvate to pyruvate^72^. Pkm2 is highly expressed in embryos and cancer cells, whereas Pkm1 is dominant in mature adult tissues^73,74^. Interestingly, despite Pkm2 having lower enzymatic activity compared to Pkm1^75^, proliferating cells engineered to express Pkm2 produce more lactate than those expressing Pkm1^76^. We found decreased Pkm1/2 ratio in mPFC of highly competitive mice. However, no difference of main glycolysis metabolites in mPFC of highly and low competitive mice was detected via metabolomics, suggesting that glycolysis did not participate in social competition regulated by Pkm AS. Besides its metabolic function, Pkm2 can promote anabolic metabolism and exert protein kinase-like effects^77^. In tumor cells, Pkm2 dimer, which had lower catalytic activity than Pkm2 tetramer, enhanced the anabolic synthesis of macromolecules through pentose phosphate pathway^78^.

Nuclear Pkm2 acts as a protein kinase to regulate gene transcription. In vitro phosphorylation analysis showed that wild type Pkm2 but not Pkm1 or kinase-dead mutant Pkm2 K367M could phosphorylate histone H3 at T11 site^79^, which plays a crucial role in tumor cell proliferation, cell-cycle progression, and the attenuation of cellular senescence. Moreover, nuclear Pkm2 was reported to phosphorylate Stat3 at Y705 site to activate transcription, which is necessary for tumor transformation and progression^38^. Here, we identified that Pkm2 could exert its kinase activity to phosphorylate GluA1 at S845 site and promote AMPA-R membrane trafficking in postsynaptic loci. Gradually increasing of Pkm2 in PL excitatory neurons of lower competitive mice mediate competition success through GluA1 phosphorylation and then orchestrates the transition from competitive success to rank elevation. And application of peptide to impede interaction between Pkm2 and GluA1 attenuated competition ability and eventually decreased social hierarchy of highly competitive mice.

**Fig. 7:**
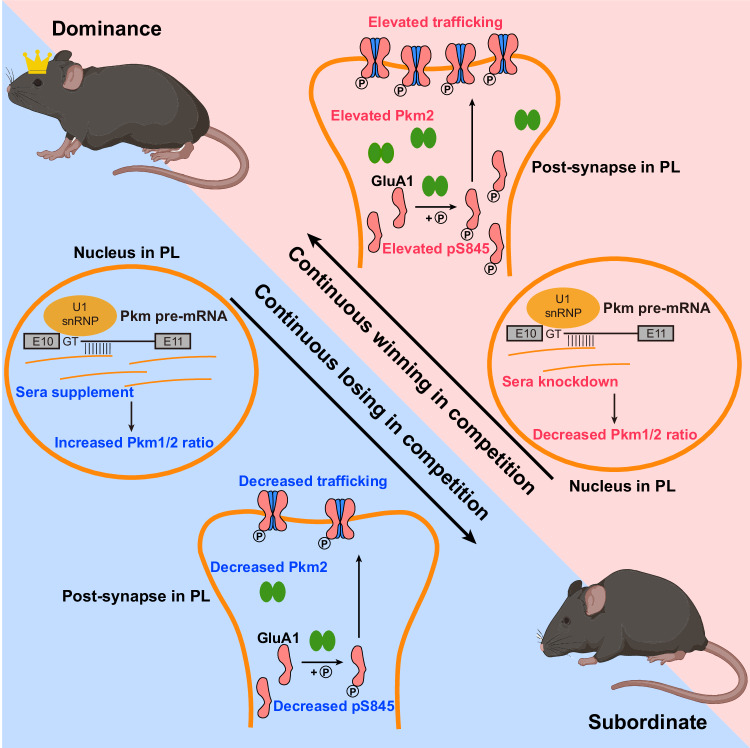
Proposed working model of lncRNA *Sera*/Pkm in social competition and rank. lncRNA *Sera* upregulation in PL of dominant mice elevates Pkm1/2 ratio and decreases social competitiveness via reducing phosphorylation of GluA1 S845. Continuous losing in competition eventually leads to decreased social rank. lncRNA *Sera* downregulation in PL of subdominant mice decreases Pkm1/2 ratio and increases social competitiveness, eventually leading to elevated social rank.

In sum, we have demonstrated a molecular mechanism that *Pkm* AS in PL excitatory neurons regulated by LncRNA *Sera* mediates AMPA-R membrane trafficking through protein kinase-like effect of Pkm2 at postsynapse, resulting in competitive ability difference within a social mouse group. We also report that Pkm2-mediated continuous competition ability alteration eventually changes social hierarchy by winner-loser effects (Fig. 7).

## Materials and methods

### Study approval

All of the animal procedures were followed by guidelines and were approved by the Animal Care and Use Committee of Tongji Medical College under approval number 2019-S855.

### Mice

Adult male C57BL/6 mice were purchased from the National Resource Center of Model Mice (Nanjing, China). Male heterozygous Pkm2 flox mice (No. 024048), CaMKIIa-CreERT2 mice (No. 012362), Ai9 mice (No. 007905) were purchased from Jackson laboratory (Bar Harbor, ME, USA). All mice were housed (four per cage) under a 12 h light/dark cycle in a temperature (22 ± 2 °C)-controlled room. They were provided with food and water ad libitum, except during food competition tests. For food competition tests, animals had free access to water but were kept at 85% of their baseline body weight. All experiments were performed on mice aged 10–12 weeks and weighed 22–25 g at the time of first experimental procedure. All procedures conformed to “Policies on the Use of Animals and Humans in Neuroscience Research” and were approved by the Institutional Animal Care and Use Committee of the Huazhong University of Science and Technology.

### Tube test

The tube test was performed following the previously reported protocol^11^. Four mice with similar age and body weights were housed in the same cage for at least 2 weeks before test. A transparent 30 × 3 cm (length × inner diameter, L × ID) tube was used for both training and testing. The diameter of the tube allowed only one adult mouse to move freely at a time. During the training phase, each mouse was trained to move forward out of the tube on each side for 10 trials (referred to as a session) in two successive days. In the testing phase, two mice entered the tube from opposite side, with their tails held, and were released when they met in the middle of the tube face to face. The mouse that forced the other one to retreat out of the tube was considered the winner of the trial. For each cage, a round robin design was used to detect hierarchy. Only groups with stable linear rank for over 3 days were selected for further competition tests.

### WS test

The WS test was performed as described previously^11^. A group of mice from the same cage were taken into a rectangular 28 × 20 cm (L × W) plastic cage which was put on ice and cooled down to 0 °C. A round nest with a diameter of 5 cm, which only permitted one adult mouse to occupy, was placed in a corner and heated by a underneath coil at 34 °C. Behaviors were video-taped for 20 min and the occupation time was analyzed. All mice used in the competition experiments exhibited a stable liner dominance hierarchy, as accessed by the tube test.

### Food competition test

Food competition test was performed according to a previous report^13,14^with minor modifications. Briefly, two starting areas, 23 × 10 × 30 cm (width × length × height, W × L × H), were attached to the outer face of a white acrylic open arena in the size of 60 × 60 × 30 cm (W × L × H). In one corner, a 19 × 19 cm area was designated as the reward zone, with a 2 × 3.5 cm (W × H) rectangular opening at bottom vertex, which allowed only one mouse to pass through at a time. Before training, mice were habituated in the arena for 15 min for three consecutive days. In training stage, each mouse was placed alternately in starting areas. After 20 s, the gate of starting area containing the mouse was open, and the mouse searched for the pellet in the reward zone. Each mouse underwent 20 successive trials per day, commencing 10 times from each of two starting areas. Mice were trained individually to forage for food pellets until they reached the criterion of reaching the reward zone with a mean latency of less than 5 s for two consecutive days. The competition tests were initiated after all mice in a cage met the learning criteria. Mice in the same cage were tested pair-wise using a round robin design. For each pair, two mice were placed in the same starting area and underwent 10 successive trials, commencing 5 times from each of two starting areas. Food pellet consumption was considered ultimate competition success.

### In vivo electrophysiology recording and analysis

After tube test and WS test, one mouse from each group, typically with mid-ranked hierarchy, was implanted with electrode. Other three mice from the same group received sham surgery. Anesthesia was induced with 3% isoflurane and maintained with 1.5% isoflurane during the surgical procedure, while a heating pad was used to stabilize body temperature. After craniotomy, three stainless steel screws were attached to the skull and one steel screw was used as a ground electrode. Mice were unilaterally implanted with electrode in PL (AP: 2.4 mm; ML: 0.25 mm DV: ‒1.8 mm). Each electrode, containing 8 stereotrodes in 2 individually insulated platinum iridium wires (100‒167, California Fine Wire Company, Grover Beach, CA, USA), was fixed to an electrode guide. All implants were secured using Super-Bond cement. After surgery, mice were allowed to recover in their home cage for 7 days. During the food competition test, PL neuronal activity was recorded. The electrodes were connected to a headstage (Plexon, Dallas, TX, USA) that featured AC-coupled unity-gain operational amplifiers (Plexon). The headstage was further connected with a 16-channel PBX preamplifier to amplify the signal by 4000- to 8000-fold and isolate the signal using a 250-Hz lowpass filter and a 250-Hz highpass filter.

An Omniplex system (Plexon) was used to isolate spiking activity through time-amplitude window discrimination and template matching. An off-line spike sorter (Plexon) was employed for single-unit spike sorting, and multivariate ANOVA statistics was used to assess unit isolation quality (*P*< 0.05). Based on physiological features, units were classified based on half width duration, area under curve (AUC), and trough-to-peak latency (TP latency). The average waveforms of recording site with maximum amplitude for the averaged waveforms of a given unit were taken to calculate TP latency, half width duration and AUC. The units were segregated into excitatory and inhibitory types within a 3D feature space using k-means clustering (k = 2). The burst index was calculated by average number of spikes in 3‒5 ms bins of spike autocorrelogram divided by calculating number of spikes in 200‒300 ms bins. Based on burst index with bimodality of the marginal distribution, the classification of excitatory and inhibitory were further validated. Recorded neurons were sorted into competition-related, rank-related, non-related and both-related neurons as previously described^13^. Notably, when we calculated Δ z-score of firing rate in rank-related neurons after Dox or peptide administration, definition of high-ranked and low-ranked mice for the recorded one followed the basal rank. At the end of the experiment, standard histological techniques were used to verify electrode tip location.

### Immunofluorescence

Immunofluorescence was performed, as described previously^80^. After anesthetized with ketamine (100 mg/kg) and dexmedetomidine (0.5 mg/kg), mice were perfused through apex cordis with 50 mL ice-cold phosphate-buffered saline (PBS), and then fixed with 50 mL 4% paraformaldehyde in PBS. After extraction, brain was post-fixed overnight in 4% paraformaldehyde. Then, brain was transferred into 30% sucrose/4% paraformaldehyde solution in PBS. Brain was cut into 30-µm coronal slices using a freezing microtome (CM1950, Leica, Wetzlar, Germany). After rinsed with PBS for 3 times (5 min each), the slices were penetrated with 0.5% Triton X-100 (T8200-50, Solarbio, Wuhan, China) for 20 min and incubated with a blocking solution of 3% bovine serum albumin (BSA) (A4503, Sigma-Aldrich, St. Louis, MO, USA)/0.1% Triton X-100 in PBS for 30 min, at the room temperature. Then, the slices were incubated with primary antibodies diluted with solution of 3% BSA/0.1% Triton X-100 in PBS at 4 °C overnight. After that, the slices were rinsed with PBS for 3 times, and then incubated with fluorescence-conjugated secondary antibodies for 1 h at room temperature. DAPI (1:500) (C0060, Solarbio) was used to visualize the nuclei. Images were captured using a Zeiss LSM800 laser confocal microscope (Zeiss, Oberkochen, Germany). The antibodies are listed in Supplementary Table S1.

### FACS-based RNA-Seq and differential splicing analysis

We followed a previously reported protocol^81^. Before food competition test, CaMKIIa-creERT2:Ai9 were intraperitoneally injected with tamoxifen (75 mg/kg) for 5 days. Highly (C1) and low (C4) competitive mice were anesthetized with ketamine (100 mg/kg) and dexmedetomidine (0.5 mg/kg). Mice were perfused through apex cordis with 50 mL ice-cold oxygenated Hank’s balanced salt solution-HEPES–trehalose. mPFC samples were extracted and cut with iris scissors. After the tissues were digested with a papain-DNase solution at 37 °C for 45 min, fetal bovine serum was added to stop the digestion process. After centrifuged at 300× *g* for 1 min, the supernatant was discarded. Dissociation solution and Percoll solution were respectively used to fully dissociate tissues and purify the cell suspension. After that, DAPI was added to cell suspension to stain dead cells. Samples were run by FACS via FacsAriaIII (BD Biosciences, New York, NY, USA). mCherry^+^cells were collected and sorted into ice-cold TRIzol (Thermo Scientific, Waltham, MA, USA), which was used to extract total RNA according to the manufacturer’s protocol.

RNA purity and quantification were assessed using the NanoDrop 2000 spectrophotometer (Thermo Scientific). RNA integrity was evaluated using the Agilent 2100 Bioanalyzer (Agilent Technologies, Santa Clara, CA, USA). The libraries were sequenced on a Novaseq 6000 platform (Illumina, San Diego, CA, USA) to generate 150 bp paired-end reads. After raw reads were processed using fastp^82^and the low-quality reads were removed, clean reads were mapped to the reference genome using HISAT2^83^. Differential splicing analysis was performed using three different tools. Both DEXSeq (Version 1.46.0)^84^and limma (Version 3.50.3)^85^are a part of the Bioconductor R package (10.18129/B9.bioc.DEXSeq), and identified AS events were based on inferring the relative exon usage within each gene. rMATS (Version 4.1.2), obtained from SourceForge (http://RNA-seq-mats.sourceforge.net/), identified AS events based on exon-included and exon-excluded junction-spanning reads^86^. The cut-off criteria were: FDR < 0.05, logFC > 2.

### Subcellular fractionantion, RNA extraction and real-time PCR

Subcellular Fractionation was performed according to a previously reported protocol^87^. After tissues were homogenized in homogenization buffer (30 mM Tris HCl pH 7.4, 225 mM mannitol, 75 mM sucrose, 0.5 mM EGTA, protease inhibitor and 0.5% BSA), the homogenate was centrifuged at 630× *g* for 5 min. Supernatant was collected as the crude cytosol. Pellet was resuspended in homogenization buffer and centrifugation was repeated twice. The supernatant was discarded and the pellet (nuclei) was used. The fractionated or total RNA from the brain tissues or cultured cells was extracted using TRIzol following the manufacturer’s instruction. A total of 1 µg RNA was used to synthesize cDNA by ReverTra RT reagent Kit (FSQ-101, Toyobo life science, Osaka, Japan). Real-time PCR was performed on ABI StepOne Plus (Thermo Scientific) using SYBR Green PCR Master Mix (C11201, Yeasen, Shanghai, China). Reaction systems were prepared including 5 µL SYBR Green, 1 μL forward primer, 1 μL reverse primer, 0.5 µL cDNA, and 2.5 µL RNase/DNase-free sterile water. Relative expression was analyzed by 2 ^−^^Δ^^Δ^^C^^t^ normalized to Actin. The primers are listed in Supplementary Table S2.

### Protein extraction and western blot

After mice were euthanized, mPFC tissues were immediately dissected. For total protein extraction, tissues were homogenized on ice with radioimmunoprecipitation assay lysis buffer (RIPA) (P0013, Beyotime, Shanghai, China). Cultured cells were also collected and lysed in RIPA. After boiling for 10 min, samples were ultrasonically disrupted on ice. For membrane protein extraction from brain tissues or cultured cells, a Mem-PER Plus Membrane Protein Extraction Kit (89842, Thermo Scientific) was used according to the manufacturer’s protocol. Protein concentration was measured by BCA Protein Assay Reagent (23225, Thermo Scientific). Note that after boiling, membrane proteins tended to aggregate and form dimers or polymers that were not well recognized by antibodies. Therefore, all the boiling steps were replaced with a water bath at 37 °C for 30 min when detecting AMPA-R and NMDA-R expressions.

Proteins were separated on 10% SDS-PAGE gels and transferred onto PVDF membranes (Bio-Rad, Hercules, CA, USA). After blocking with 5% non-fat milk for 1 h, the membranes were incubated with primary antibodies overnight at 4 °C, followed by secondary antibody (800 R or 800 M, LI-COR, Lincoln, NE, USA) incubation for 1 h. The protein bands were visualized using the Odyssey Imaging System (LI-COR). ImageJ software was used to quantify intensity of the bands. Absolute values were normalized to loading control (Actin for total proteins, and Atp1a1 for membrane proteins) bands from the same samples. Primary antibodies are listed in Supplementary Table S1.

### Virus injection and Dox application

Virus injection was performed as described previously^88^. After anesthetized with ketamine (100 mg/kg) and dexmedetomidine (0.5 mg/kg), mice were mounted by stereotaxic apparatus (RWD life science, Shenzhen, China). 300 nL of virus for each site was injected into PL (AP: 2.4 mm; ML: ±0.25 mm DV: ‒1.8 mm), at an infusion rate of 0.2 μL/min using a Hamilton microsyringe. After injection, the needle was left in brain for 10 min and slowly withdrawn. Before behavioral tests, mice were allowed to recover for 3–4 weeks in their home cage. To activate virus containing a tet-on/off system, mice were intraperitoneally injected with Dox (7 mg/Kg) for consecutive 21 days. At the end of the experiment, we confirmed the injection sites and excluded mice with incorrect injection placement. siRNAs for *Pkm1*, *Pkm2* and *Sera* were designed by BLOCK-iT™ RNAi Designer and the efficiency was validated in N2a cells. Plasmids and virus were purchased from ObiO Technology (Shanghai, China). The sequences of siRNAs and primers used for constructing overexpression plasmids are listed in Supplementary Table S3.

### Cross-correlation analysis

To evaluate for temporal changes in relation to Dox or peptide administration, we used Δ z-score of firing rate as the neuronal point data, and the proportion of winning trials as the behavioral point data. Cross-correlation analysis was used to assess the temporal relationship between neuronal and behavioral data, as previously reported^89^.

### FISH

Mice were perfused with 0.9% NaCl and 4% PFA. After post-fixation and dehydration, brains were cut into 20-µm coronal slices with freezing microtome. Slices were permeabilized in 0.5% Triton X-100 for 15 min, and blocked by pre-hybridization buffer at 37 °C for 30 min. Hybridization was carried out in a moist chamber at 37 °C for over 12 h in the dark, using Ribo™ Fluorescent In Situ Hybridization Kit (RiboBio, Guangzhou, China). Then, slices were rinsed with 4× SSC for three times, 2× SSC, and 1× SSC in the dark. Afterwards, the brain slices were blocked with 3% BSA for 1 h and stained with primary antibody overnight at 4 °C. After slices were incubated with fluorescent dye-conjugated secondary antibody for 1 h, DAPI was used to stain the nuclei for 10 min. Images were captured using a Zeiss LSM800 laser confocal microscope. FISH probes were synthesized by Tsingke Biotech, Beijing, China.

### Cell culture and transfection

Mouse N2a cells were cultured in Dulbecco’s modified Eagle’s medium (DMEM) (Thermo Scientific) supplemented with 10% fetal bovine serum (FBS), (MK1124, MIKX, Shenzhen, China) and maintained at 37 °C in 5% carbon dioxide (CO_2_). Primary neurons were isolated from the cortices of C57 mice as described previously^88^. The cortices of embryos were dissected on embryonic day 16 (E16), and the meninges were removed. After digestion in trypsin at 37 °C for 10 min, tissues were filtered through a 40-μm cell strainer. Then, the collected neurons were placed in six-well plates and incubated for 3 h with plating medium of DMEM/F12 (Thermo Scientific) supplemented with 10% FBS and 1% penicillin/streptomycin. Afterwards, the plating medium was replaced with maintenance medium (Neurobasal medium with 2% B-27, 1× GlutaMAX, and 1% penicillin/streptomycin). For virus infection of primary neurons, AAVs were added on day 7 in vitro (DIV7), and the media were fully replaced on DIV15. Transfection of plasmids or AMO was carried out with Lipofectamine 3000 (Thermo Scientific) according to the manufacturer’s instructions. The AMO and its relative control were synthesized by RiboBio.

### Rapid amplification of cDNA ends (RACE) of full-length *Sera*

RNA extraction from C57 mouse brains was prepared using TRIzol. 5′ and 3′ RACE cDNA libraries were synthesized using SMARTer RACE 5′/3′ Kit (Takara, Beijing, China). RACE amplification products were analyzed with agarose gel electrophoresis and Sanger sequencing. The primers were listed in Supplementary Table S2.

### RIP

RIP was performed as previously described^90^. Cells were lysed on ice with RIP buffer containing 5 mM MgCl_2_,100 mM KCl,10 mM HEPES, 0.5% NP-40, 1 mM Dithiothrectol, RNase and protease inhibitors for 30 min. After cell debris was removed by centrifugation, cell lysate was pre-bound with protein G beads (Thermo Scientific) for 2 h at room temperature. Anti-Snrpa antibody (10212-1-AP, Proteintech, Wuhan, China) was used to pull down the binding RNAs. After co-immunoprecipitated RNAs were extracted using TRIzol, cDNA was synthesized with the ReverTra RT reagent Kit and analyzed by real-time PCR. Amount of immunoprecipitated RNAs was compared to the input. The primers used in RIP-qPCR were listed in Supplementary Table S2.

### Metabonomics and analysis

After mPFC samples were dissected from highly and low competitive mice, three-phase solvent system including MTBE, methanol and water was used to extract metabolites. Non-targeted metabolomics were carried out using Ultimate 3000 High Performance Liquid Chromatograph (Thermo Scientific). and the Q Exactive quadrupole-Orbitrap Mass Spectrometer System (Thermo Scientific). The medium polar metabolites were detected by positive and negative ion detection modes, and polar metabolites were isolated by hydrop interaction liquid chromatography. Compound Discoverer software (Thermo Scientific) was used to process full-scan and data-dependent metabolic profile for comprehensive component extraction. XCalibur Quan Browser (Thermo Scientific) was used to extract the AUCs of the total ion flow diagram. The quantitative information of metabolites was combined for the final statistical analysis. Multivariate data were analyzed by SIMCA-P software (Umetrics, Umea, Sweden). Univariate analyses including independent sample *t*-tests and *P*-value FDR adjustments, as well as metabolic enrichment analyses, were performed on the MetaboAnalyst.

### Co-immunoprecipitation and mass spectrum

Mouse cerebral cortex was homogenized on ice with RIPA lysis and proteinase inhibitor mixture. After clearing debris by centrifugation, protein concentration was quantified with BCA Protein Assay Reagent. Total extracted proteins were incubated with anti-Pkm2 antibody (1:50) overnight. Rabbit IgG (30000-0-AP, Proteintech) was used as a negative IP control. The mixtures were incubated with beads for 4 h, and washed 4 times. IP sample and input control were boiled with SDS buffer for 10 min. After proteins were separated on 10% SDS–PAGE gels, gels were stained with Coomassie Brilliant Blue R250 (P0017B, Beyotime) for 2 h at room temperature. After washed with Coomassie Blue Staining Destaining Solution (P0017C, Beyotime) for 4 times overnight, protein bands were cut and pre-processed with acetonitrile. Samples were separated with UltiMate 3000 RSLCnano (Thermo Scientific), and the mass spectrometry was performed using Q Exactive Plus LCMS system (Thermo Scientific). The mass spectrum data were retrieved by MaxQuant (V1.6.2.10) using MaxLFQ algorithm. For phosphorylation site identification, a sample of purified GluA1 that was in vitro phosphorylated by Pkm2 was used. Phosphopeptide matches were analyzed by using PhosphoRS implemented in Proteome Discoverer.

### Whole-cell patch-clamp recording

Whole-cell patch-clamp recording was performed as previously described^91^. For mEPSC recordings, neurons were recorded at −70 mV, using a pipette solution containing 140 mM potassium gluconate, 2 mM NaCl, 0.2 mM EGTA, 10 mM HEPES, 2 mM MgATP, and 0.3 mM NaGTP. The external solution contained 10 µM bicuculline, 1 µM tetrodotoxin and 50 µM APV. The electrophysiological data were filtered at 2 kHz and acquired at 10 kHz (Molecular Devices, Sunnyvale, CA, USA). mEPSC events were analyzed using MiniAnalysis (Synaptosoft, Decatur, GA, USA), under the detection criteria of an amplitude greater than 5 pA, a minimum rise rate of 0.3 pA/ms, and a decay time constant between 1 and 12 ms. The data were analyzed using GraphPad Prism.

### Crude synaptosomal preparation

Mouse cerebral cortex was homogenized on ice with HEPES-buffered sucrose (pH 7.4) containing 0.32 M sucrose, 4 mM HEPES, and protease inhibitor cocktail. Samples were centrifuged at 1000× *g* for 10 min to extract nuclear enriched pellet and the S1 fraction. S1 fraction was centrifuged at 12,000 × *g* for 20 min to separate S2 supernatant (microsomes and cytosol) and P2 pellet (crude synaptosomal membranes). The P2 pellet was resuspended in HEPES buffer (pH 7.4) containing 4 mM HEPES and 1 mM EDTA, and then centrifuged at 12,000 × *g*for 20 min. Resuspension and centrifugation were repeated twice. Afterwards, pellet was resuspended in buffer A (pH 7.2) containing 20 mM HEPES, 100 mM NaCl, and 0.5% Triton, and rotated slowly. After centrifugation at 12,000 g for 20 min, supernatant containing peri-/extrasynaptic and presynaptic membranes was separated. The pellet was resuspended in buffer B (pH 7.5) containing 20 mM HEPES, 0.15 mM NaCl, 1% Triton-X, 1% deoxycholic acid, 1% SDS, and 1 mM DTT, and was slowly rotated for 1 h. After centrifugation at 10,000 × *g*for 15 min, final supernatant contained PSD fraction.

### Immunoelectron microscope

Immunoelectron microscope was performed, as previously described^92^. 4% paraformaldehyde was used for mouse brain fixation at 4 °C for 1 h. After brain was cut into 40-µm coronal slices using a vibrating blade microtome (VT1000S, Leica), 1% glutaraldehyde was used for sample fixation at 4 °C for 2 h. Brain slices containing mPFC were rinsed with 0.1 M PBS for 4 times (20 min each), and then penetrated with 0.5% Triton X-100 for 20 min. After blocked by 3% BSA for 15 min, slices were incubated with anti-Pkm1 (1:20) or anti-Pkm2 (1:20) antibody overnight at 4 °C, and then incubated with 10 nm colloidal gold secondary antibody (A-31566, Thermo Scientific) overnight at 4 °C. After fixed with 3% glutaraldehyde for 30 min and 1% osmic acid for 1.5 h, slices were dehydrated in 30%, 50%, 70%, 90%, and 100% ethanol, and were incubated with propylene oxide twice (15 min each), Epon: propylene oxide (1:2) for 1 h, and Epon: propylene oxide (4:1) overnight. Afterwards, slices were polymerized with Epon at room temperature for 2 h, and then at 60 °C for 2 days. Samples were ultrathin-sectioned into 50 nm for transmission electron microscope (HT-7700, Hitachi, Tokyo, Japan).

### GST-tagged protein purification and in vitro phosphorylation assay

pGEX plasmids expressing GST-Pkm1, GST-Pkm2, or GST-GluA1 were transformed into BL21(DE3) competent cells (B528414, Sangon Biotech, Shanghai, China). IPTG was added to induce protein expressions. Bacteria were collected through centrifugation at 4200× *g* for 15 min. With lysozyme and proteinase inhibitor added, bacteria were ultrasonically disrupted on ice. GST-tagged protein purification was performed using GST-tag Protein Purification Kit (P2262, Beyotime) according to the manufacturer’s instructions. In vitro phosphorylation assay was performed as described previously^75^. 200 ng purified Pkm1 or Pkm2 from bacterial system were incubated with GluA1 with kinase buffer containing 50 mM Tris-HCl (pH 7.5), 100 mM KCl, 50 mM MgCl_2_, 1 mM Na_3_VO_4_, 1 mM PMSF, 2 mM DTT, 2 mM PEP, 0.5 mM FBP and 5 mM ATP, in 30 μL at 30 °C for 1 h. The reactions were terminated by the addition of SDS-PAGE loading buffer. The reaction mixtures were then subjected to SDS-PAGE analyses.

### Administration of peptides

Membrane-permeable blocking peptides (TLPRNSGAGAS-TAT) were applied in primary cultured neurons in a concentration gradient of 0, 0.25, 0.5, 1, 2 or 4 μM. Peptides (10 mg/kg) or saline were intraperitoneally injected into highly competitive mice for 21 days. The peptides with 95.79% purity were synthesized by ChinaPeptides (Shanghai, China).

### Quantification and statistical analysis

All data were shown as means ± SEM. Data were analyzed using GraphPad Prism (GraphPad Software, San Diego, CA, USA), SPSS (IBM, Armonk, New York, USA), R Studio or MATLAB (Mathworks, Natick, MA USA). A paired Student’s *t*-test was used to assess the variance between two groups. Two-way ANOVA analysis was used to assess difference among multiple groups. Correlations were assessed by linear regression (χ^2^ test). *P* < 0.05 was taken to indicate statistical significance. *P < 0.05, **P < 0.01, ***P < 0.001, and ****P < 0.0001.

## Supplementary information

Supplementary figures

## Data Availability

All data associated with this study are present in the paper or Supplementary Materials. The software used in the current study has been cited in Materials and Methods. Please address all requests for reagents and materials to L.-Q.Z. (zhulq@mail.hust.edu.cn).
