# Author Correction: Postsynaptic lncRNA *Sera*/Pkm2 pathway orchestrates the transition from social competition to rank by remodeling the neural ensemble in mPFC

**DOI:** 10.1038/s41421-025-00782-4

**Published:** 2025-03-14

**Authors:** Ling-Shuang Zhu, Chuan Lai, Chao-Wen Zhou, Hui-Yang Chen, Zhi-Qiang Liu, Ziyuan Guo, Hengye Man, Hui-Yun Du, Youming Lu, Feng Hu, Zhiye Chen, Kai Shu, Ling-Qiang Zhu, Dan Liu

**Affiliations:** 1 Department of Pathophysiology, Key Lab of Neurological Disorder of Education Ministry, School of Basic Medicine, Tongji Medical College, Huazhong University of Science and Technology, Wuhan, Hubei China; 2 Center for Stem Cell and Organoid Medicine (CuSTOM), Division of Developmental Biology, Cincinnati Children’s Hospital Medical Center, Cincinnati, OH USA; 3 Department of Biology, Boston University, Boston, MA USA; 4 Department of Neurosurgery, Tongji Hospital, Tongji Medical College, Huazhong University of Science and Technology, Wuhan, Hubei China

**Keywords:** Non-coding RNAs, Membrane trafficking

Correction to: *Cell Discovery* (2024) **10**(1):87 10.1038/s41421-024-00706-8, published online 20 August 2024

In the original publication of this article, we inadvertently replaced the Pkm2/*AK018848*/DAPI staining images for the IL region of the C4 mouse group in Supplementary Fig. S12d with incorrect images. These incorrect images were taken from the same brain slide as the Pkm1/*AK018848*/DAPI staining images for the PL region of the C1 mouse group in Supplementary Fig. S12c.

Additionally, while we typically apply the same parameters for imaging the same brain region and staining across different experimental groups (e.g., C1 and C4), the images in Supplementary Figs. S12c and S12d were processed with different parameters, resulting in noticeable discrepancies in fluorescence intensity and image scaling.

The correct Supplementary Fig. S12d is displayed as below. This correction does not affect the results or the conclusion of this work. We are sorry for any inconvenience that might cause.
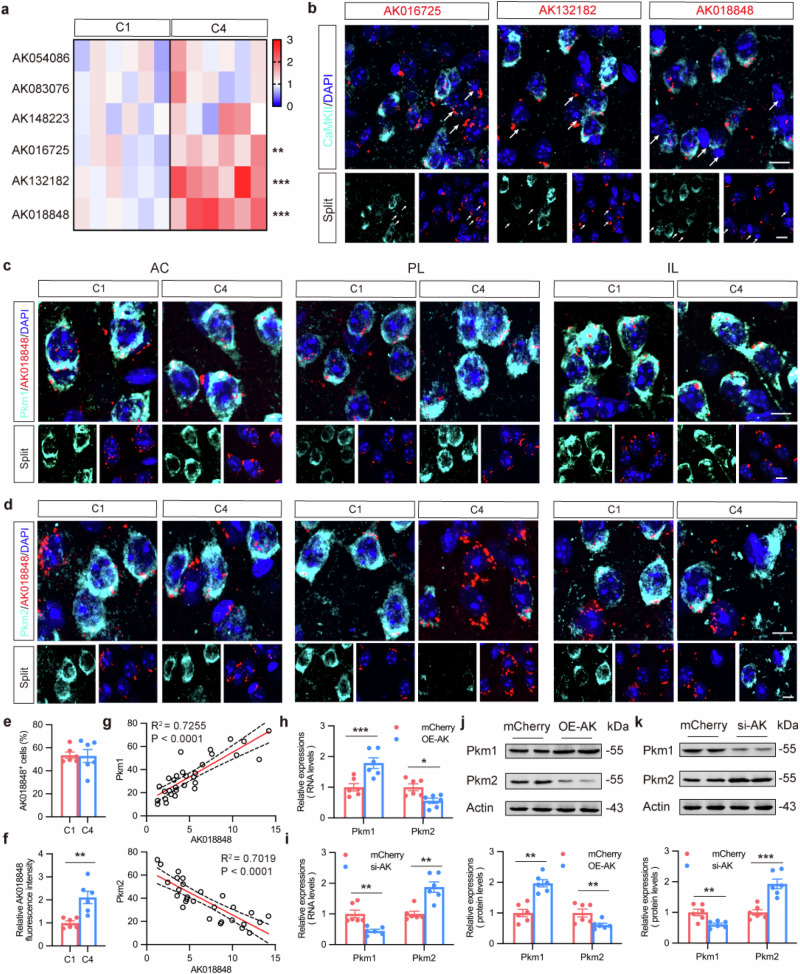


**Supplementary Fig. S12**. ***AK018848*** **mediates alternative splicing of** ***Pkm***.

**a** LncRNA expression in mPFC of C1 and C4 mice detected by qPCR. *n* = 6.

**b** Representative double-immunofluorescence images with antibodies against CaMKII (turquoise) and FISH for *AK016725*, *AK132182* or *AK018848* (red) in PL. Bar = 30 μm.

**c, d** Representative double-immunofluorescence images with antibodies against Pkm1 or Pkm2 (turquoise) and FISH for *AK018848* (red) in mPFC. Bar = 30 μm.

**e** Lack of difference in proportion of *AK018848*^+^ Cells. *n* = 6.

**f** Quantification for relative fluorescence intensity of *AK018848* in PL. *n* = 6.

**g** Correlation of fluorescence intensity between *AK018848* and Pkm1 or Pkm2.

**h, i** Relative mRNA levels of Pkm1 and Pkm2 via qPCR after AAV-CMV-AK018848-mCherry or AAV-CMV-si-AK018848-mCherry application on mouse hippocampal primary neurons. *n* = 6.

**j, k** Representative blots (up) and quantification (down) of Pkm1 and Pkm2 protein after AAV-CMV-AK018848-mCherry or AAV-CMV-si-AK018848-mCherry was applied on mouse hippocampal primary neurons. *n* = 6.

All data are shown as means ± SEM. For **a**, **e** and **f**, paired *t*-test was used. For **h**–**k**, unpaired *t*-test was used. For **g**, simple linear regression was used. *P < 0.05, **P < 0.01, ***P < 0.001.

