# Supplementary material for: Postsynaptic lncRNA Sera/Pkm2 pathway orchestrates the transition from social competition to rank by remodeling the neural ensemble in mPFC
Source: Cell Discov. 2024 Aug 20;10:87. doi: 10.1038/s41421-024-00706-8 (PMC11333582; doi:10.1038/s41421-024-00706-8)
Supplement: Supplementary file 1 — Supplementary figures [file 41421_2024_706_MOESM1_ESM.pdf]

Supplementary figures

Supplementary Fig. S1

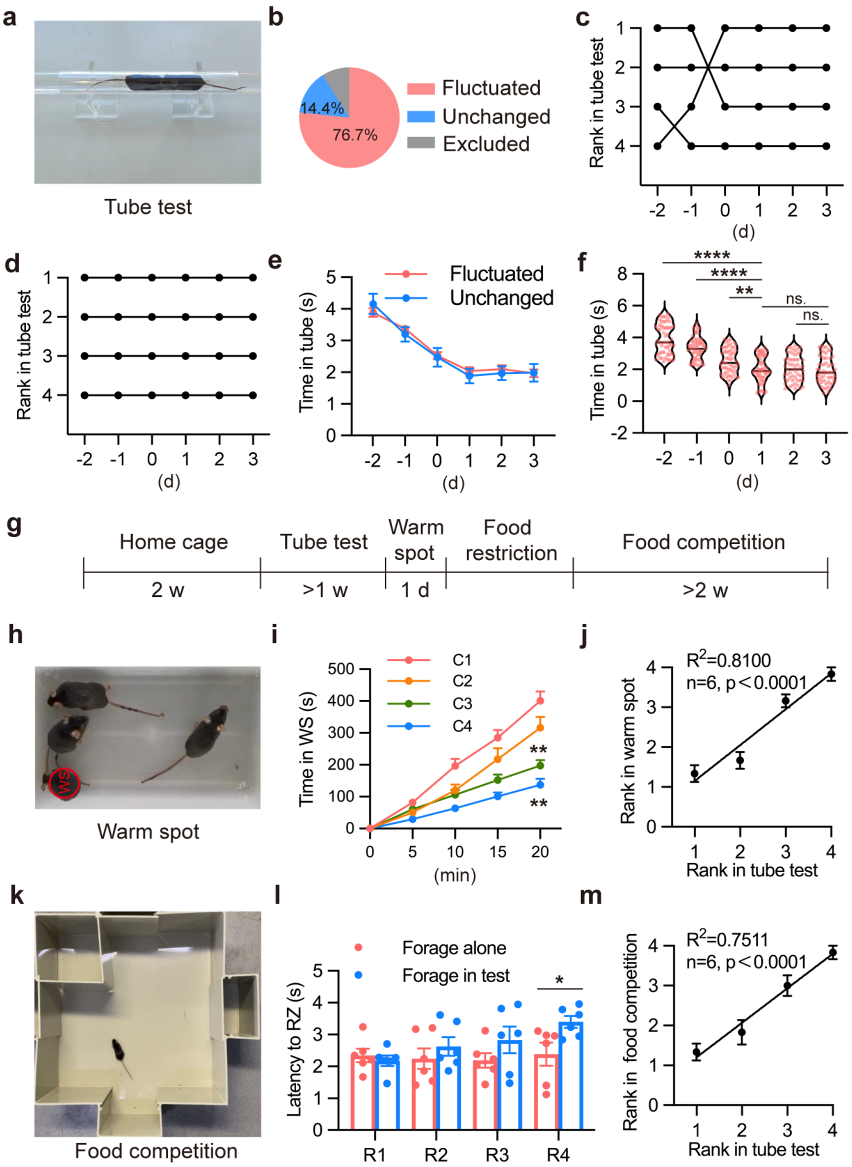

Supplementary Fig. S1. Behavior tests of social group mice.

a Representative image of tube test.

b Proportion of social groups. *n* = 90.

c Example of the rank positions of one fluctuated group.

d Example of the rank positions of one unchanged group.

e Lack of difference in average time in the tube test between unchanged groups (*n* = 13) and fluctuated groups (*n* = 69).

f Average time in the tube test for 6 days. *n* = 82.

g Experimental diagram of behavioral tests

h, **i** Representative image and quantification in the warm spot test. *n* = 6.

j Correlation of rank in the tube test and rank in warm spot test.

k Representative image of food competition training.

l Average latency to reach RZ when mice were foraged alone or in test. Each plot represents a mouse. n = 6.
m Correlation of rank in the tube test and rank in food competition test.
All data are presented as means ± SEM. For **e** and **i**, two-way analysis of variance (ANOVA) with Bonferroni analysis was used. For **f**, two-way ANOVA with Dunnett's multiple-comparison test was used. For **j** and **m**, simple linear regression was used. For **l**, paired *t*-test was used. $^*P < 0.05$, $^{**}P < 0.01$, $^{***}P < 0.001$, and $^{****}P < 0.0001$.

Supplementary Fig. S2

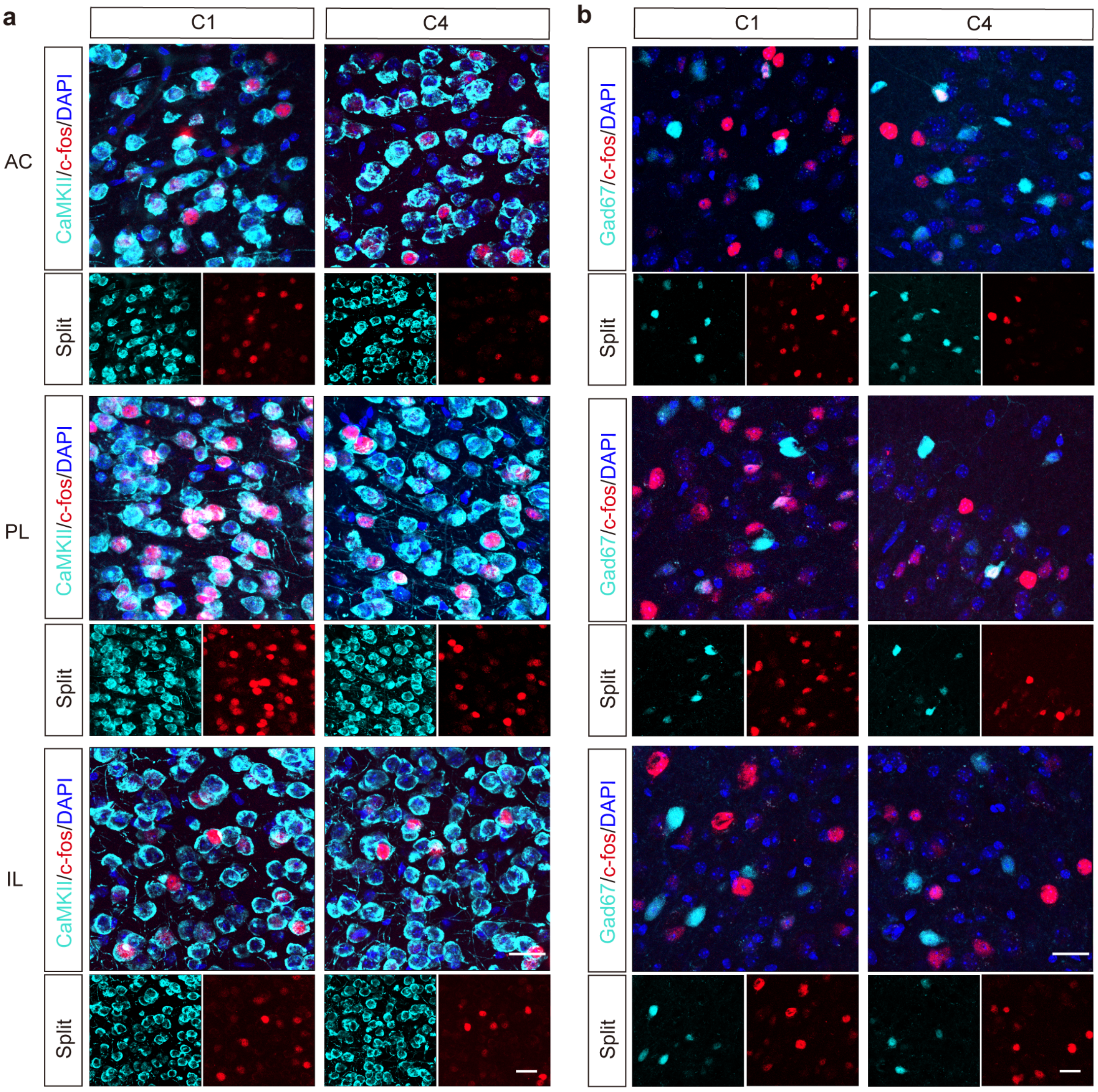

Supplementary Fig. S2. c-fos staining in mPFC excitatory and inhibitory neurons.

a Representative double-immunofluorescence images with antibodies against c-fos (red) and CaMKII (turquoise) in AC, PL and IL of C1 and C4 mice. Bar = 50 μm.

b Representative double-immunofluorescence images with antibodies against c-fos (red) and Gad67 (turquoise) in AC, PL and IL of C1 and C4 mice. Bar = 50 μm.

Supplementary Fig. S3

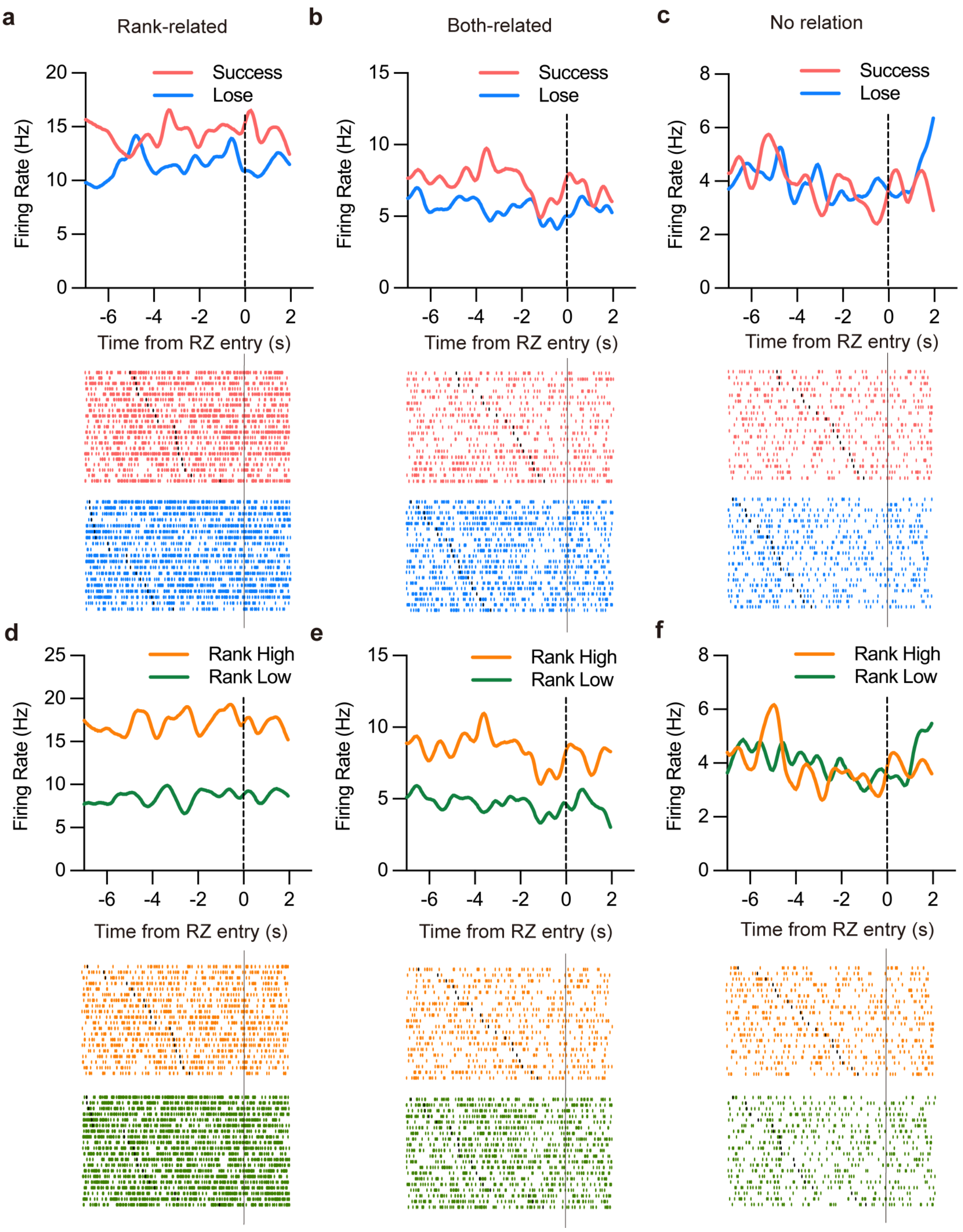

Supplementary Fig. S3. Classification of recorded neurons in PL.
Peri-event histogram and spike raster plots of rank-related, both-related, and no relation neurons showing changes in firing rate based on competitive success (**a-c**) and relative rank (**d-f**).

Supplementary Fig. S4

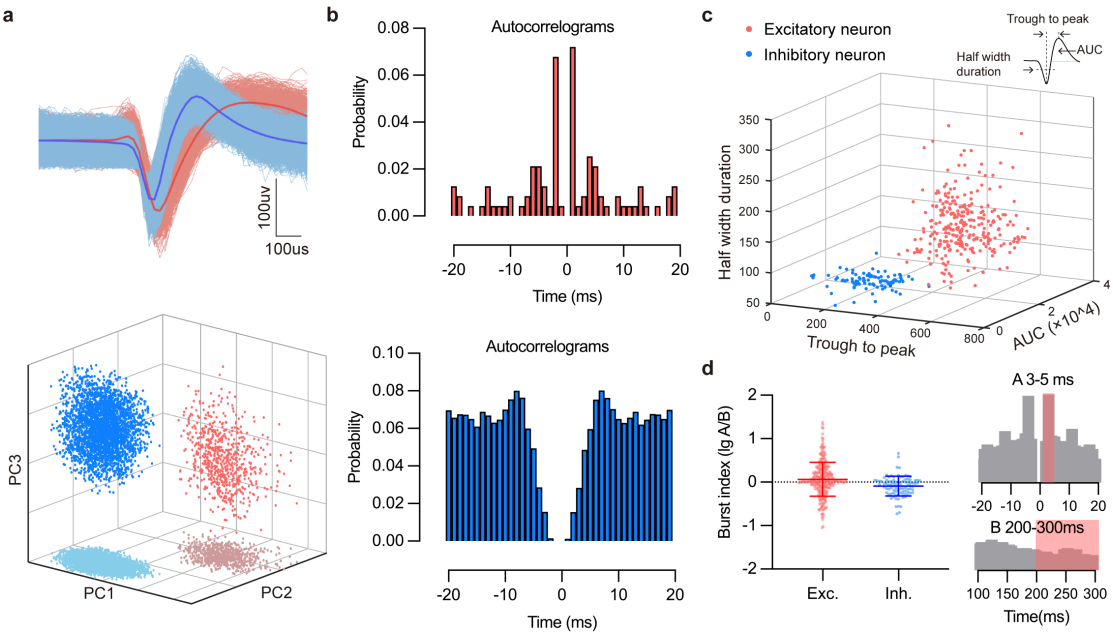

Supplementary Fig. S4. Classification of recorded neurons in PL based on physiological properties.

a, **b** Example of single unit recorded in PL showing the average waveforms (**a**, up), isolation in the principal component (PC) space (**a**, down) and autocorrelograms (**b**).

c Trough to peak distance, area under the curve (AUC) and Half width duration were used to separate units into excitatory and inhibitory, using *k*-means clustering.

d Burst index of excitatory and inhibitory neurons. Right, number of spikes in the 3–5 ms bins divided by the average number of spikes in the 200-300 ms bins.

Supplementary Fig. S5

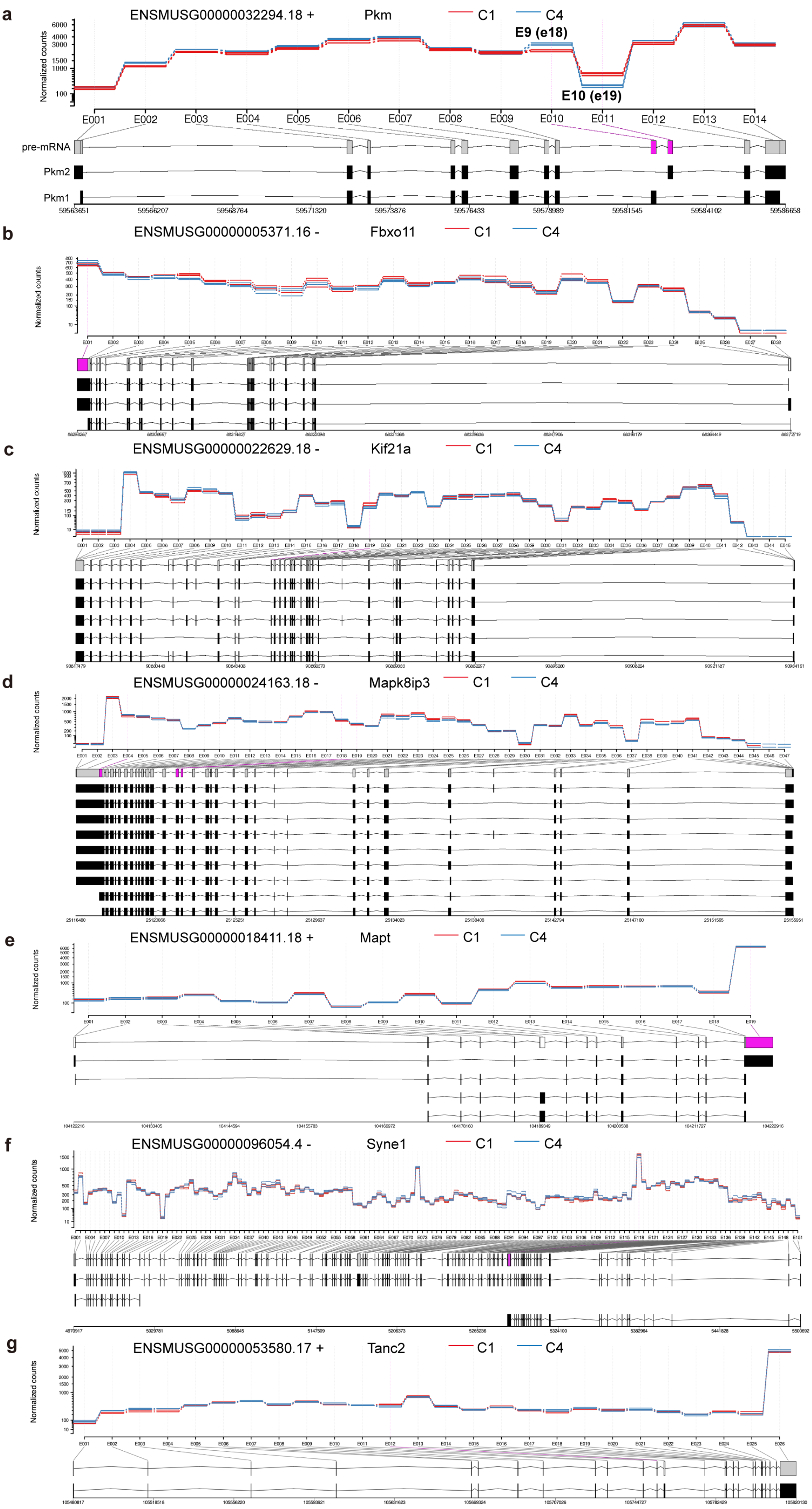

Supplementary Fig. S5. Seven genes with differential splicing events analyzed with DEXseq.

Up: normalized counts for each sample; down: flattened gene models. Differentially spliced exons were marked in pink.

Supplementary Fig. S6

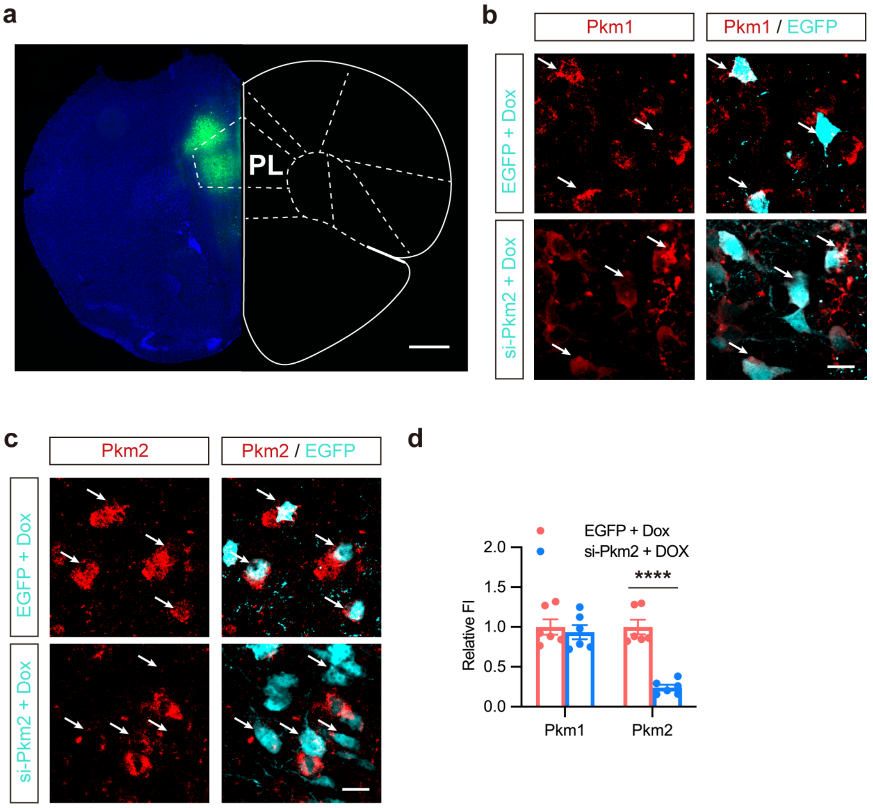

Supplementary Fig. S6. Pkm2 knockdown decreases Pkm2 expression in C1 or C2 mice.

a Representative image of EGFP fluorescence after virus injection. Bar = 500 μm.

b, c Representative immunofluorescence images with EGFP (turquoise) and antibody against Pkm1 (red) or Pkm2 (red) in PL. Bar = 50 μm.

d Quantification for relative fluorescence intensity of Pkm1 and Pkm2 in EGFP$^+$ cells of PL. *n* = 6.

All data are presented as means ± SEM, and unpaired *t*-test was used for analysis. ****$P < 0.0001$.

Supplementary Fig. S7

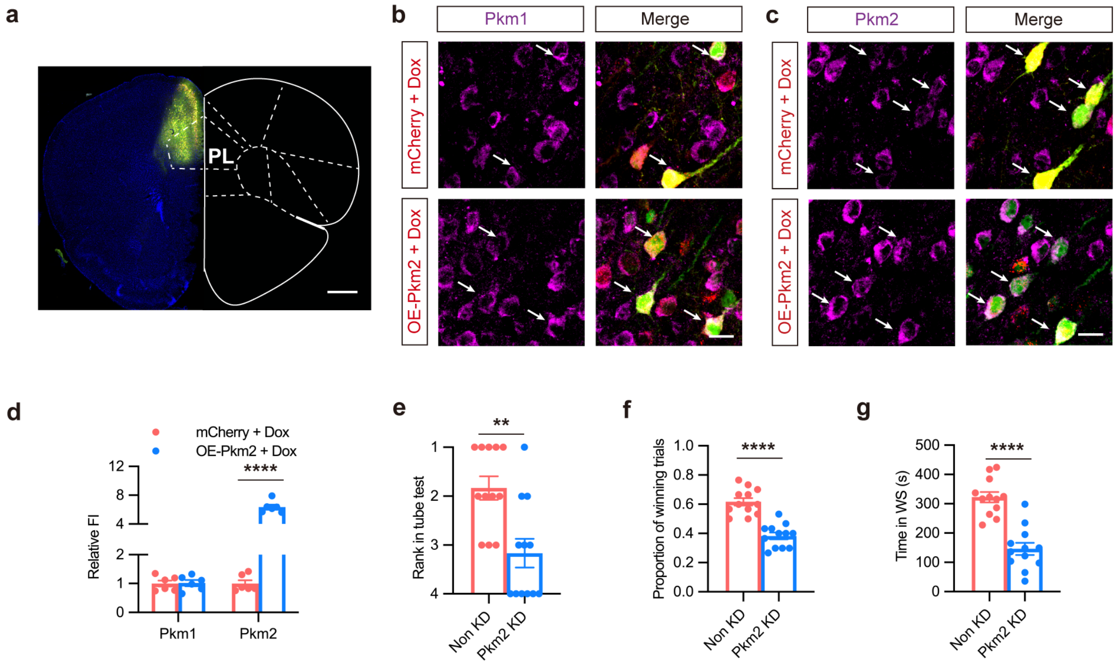

Supplementary Fig. S7. Pkm2 supplement in Pkm2 KD mice increases the Pkm2 expression.

a Representative image of EGFP and mCherry fluorescence after virus injection. Bar = 500 μm.

b, c Representative immunofluorescence images with EGFP, mCherry and antibody against Pkm1 (purple) or Pkm2 (purple) in PL. Bar = 50 μm.

d Quantification for relative fluorescence intensity of Pkm1 and Pkm2 in mCherry^{+} EGFP^{+} cells of PL. *n* = 6.

e Average rank of Pkm2 KD and non KD mice in tube test. *n* =12 from 6 groups.

f Average proportion of winning trials in food competition test. *n* = 12 from 6 groups.

g Average occupation time of warm spot in warm spot test. *n* = 12 from 6 groups.

All data are presented as means ± SEM, and unpaired *t*-test was used for analysis. $^{**}P < 0.01$ and $^{****}P < 0.0001$.

Supplementary Fig. S8

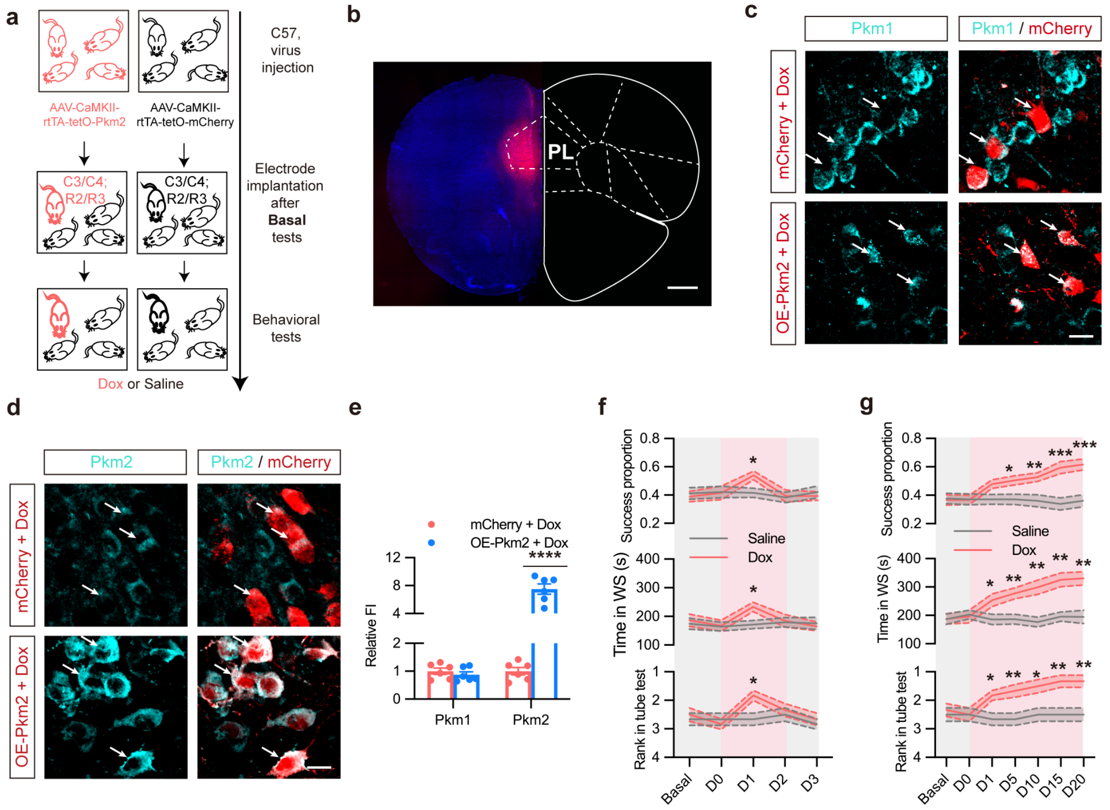

Supplementary Fig. S8. Pkm2 supplement in C57 C3 or C4 mice increases competitiveness and rank.

a Schematic illustration of experiment.

b Representative image of mCherry fluorescence after virus injection. Bar = 500 μm.

c, d Representative immunofluorescence images with mCherry and antibody against Pkm1 (turquoise) or Pkm2 (turquoise) in PL. Bar = 50 μm.

e Quantification for relative fluorescence intensity of Pkm1 and Pkm2 in mCherry^{+} cells of PL. *n* = 6.

f, g Behavioral recordings of low competitive C57 mice injected with AAV-CaMKII-rtTA-tetO-Pkm2-mCherry, and applied with single-dose Dox (**f**) or Dox for 21 days (**g**). Top: winning proportions of pellet consumption in food competition test, *n* = 18 sessions from 6 groups; middle: occupation time of warm spot in warm spot test, *n* = 6 from 6 groups; bottom: rank in tube test, *n* = 6 from 6 groups.

All data are presented as means ± SEM. For **e**, unpaired *t*-test was used. For **f** and **g**, two-way ANOVA with Dunnett's multiple-comparison test was used. *$P < 0.05$, **$P < 0.01$, ***$P < 0.001$, and ****$P < 0.0001$.

Supplementary Fig. S9

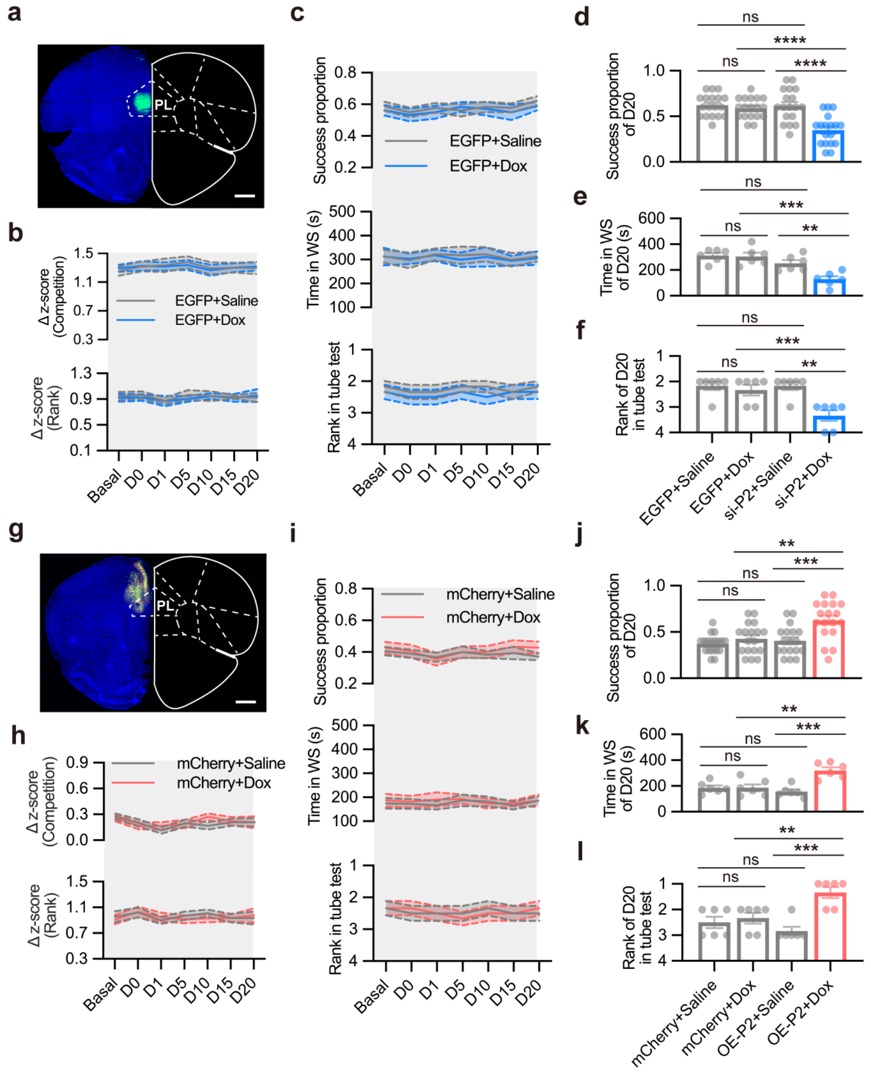

Supplementary Fig. S9. Dox treatment and the control virus did not affect social competition and rank in highly competitive mice and Pkm2 KD mice

a Representative image of EGFP fluorescence after virus injection. Bar = 500 μm.

b Neuronal recordings. Up: Δ z-score of competition-related neuron firing rate; down: Δ z-score of rank-related neuron firing rate.

c Behavioral recordings of highly competitive C57 mice injected with AAV-CaMKII-rtTA-tetO-siRNA-EGFP. Top: winning proportions of pellet consumption in food competition test, *n* = 18 sessions from 6 groups; middle: occupation time of warm spot in warm spot test, *n* = 6 from 6 groups; bottom: rank in tube test, *n* = 6 from 6 groups.

d Winning proportions of pellet consumption in food competition test on day 20, *n* = 18 sessions from 6 groups.

e Occupation time of warm spot in warm spot test on day 20, *n* = 6 from 6 groups.

f Rank in tube test on day 20, *n* = 6 from 6 groups.

g Representative image of EGFP and mCherry fluorescence after virus injection. Bar = 500 μm.
h Neuronal recordings. Up: Δ z-score of competition-related neuron firing rate; down: Δ z-score of rank-related neuron firing rate.
i Behavioral recordings of Pkm2-flox mice injected with AAV-CaMKII-cre-EGFP and AAV-CaMKII-rtTA-tetO-mCherry. Top: winning proportions of pellet consumption in food competition test, n = 18 sessions from 6 groups; middle: occupation time of warm spot in warm spot test, n = 6 from 6 groups; bottom: rank in tube test, n = 6 from 6 groups.
j Winning proportions of pellet consumption in food competition test on day 20, n = 18 sessions from 6 groups.
k Occupation time of warm spot in warm spot test on day 20, n = 6 from 6 groups.
l Rank in tube test on day 20, n = 6 from 6 groups.
All data are presented as means ± SEM. For **b**, **c**, **h** and **i**, two-way ANOVA with Bonferroni analysis was used. For **d**, **f**, **j** and **l**, two-way ANOVA with Dunnett's multiple-comparison test was used. **$P < 0.01$, ***$P < 0.001$, and ****$P < 0.0001$.

Supplementary Fig. S10

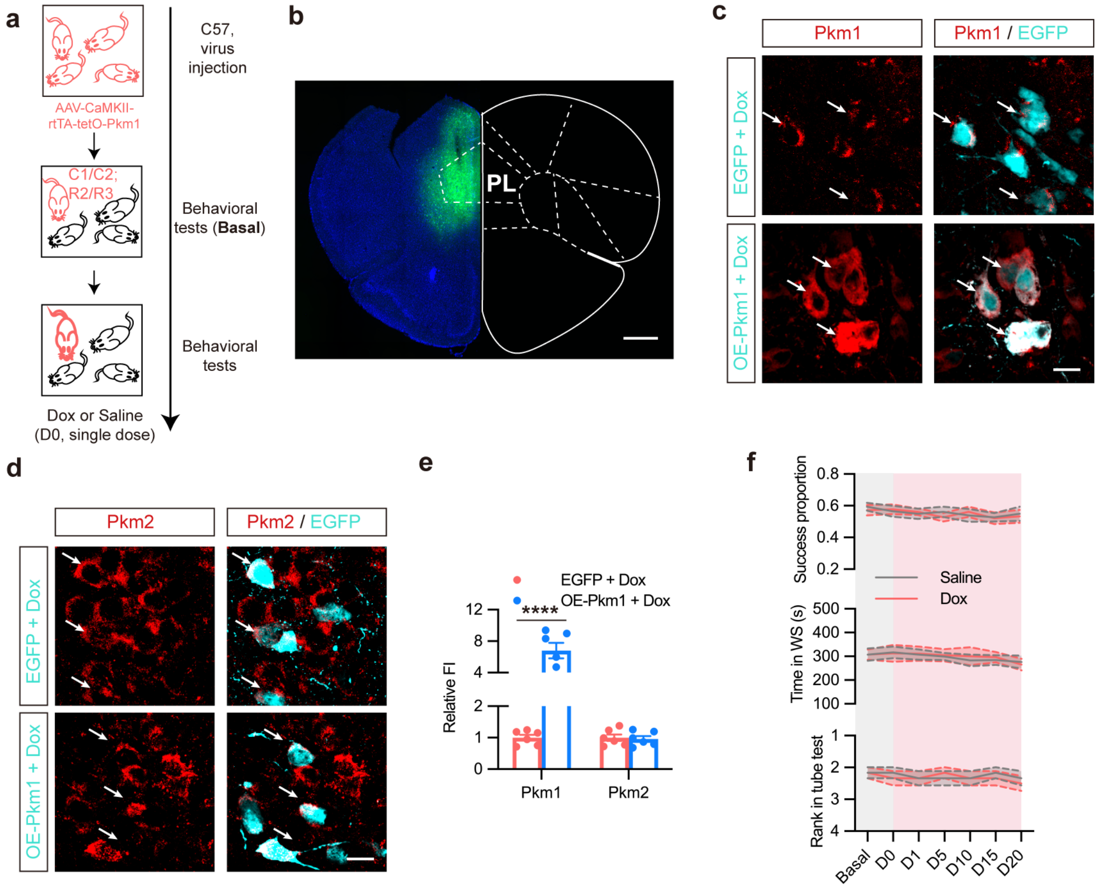

Supplementary Fig. S10. Pkm1 supplement in PL of C1 or C2 mice does not mediate competition behavior.

a Schematic illustration of experiment.

b Representative image of EGFP fluorescence after virus injection. Bar = 500 μm.

c, b Representative immunofluorescence images with EGFP (turquoise) and antibody against Pkm1 (red) or Pkm2 (red) in PL. Bar = 50 μm.

e Quantification for relative fluorescence intensity of Pkm1 and Pkm2 in EGFP$^+$ cells of PL. *n* = 6.

f Behavioral recordings of highly competitive C57 mice injected with AAV-CaMKII-rtTA-tetO-Pkm1-EGFP. Top: winning proportions of pellet consumption in food competition test, *n* = 18 sessions from 6 groups; middle: occupation time of warm spot in warm spot test, *n* = 6 from 6 groups; bottom: rank in tube test, *n* = 6 from 6 groups.

All data are presented as means ± SEM. For **e**, unpaired *t*-test was used. For **f**, two-way ANOVA with Dunnett's multiple-comparison test was used. ****$P < 0.0001$

Supplementary Fig. S11

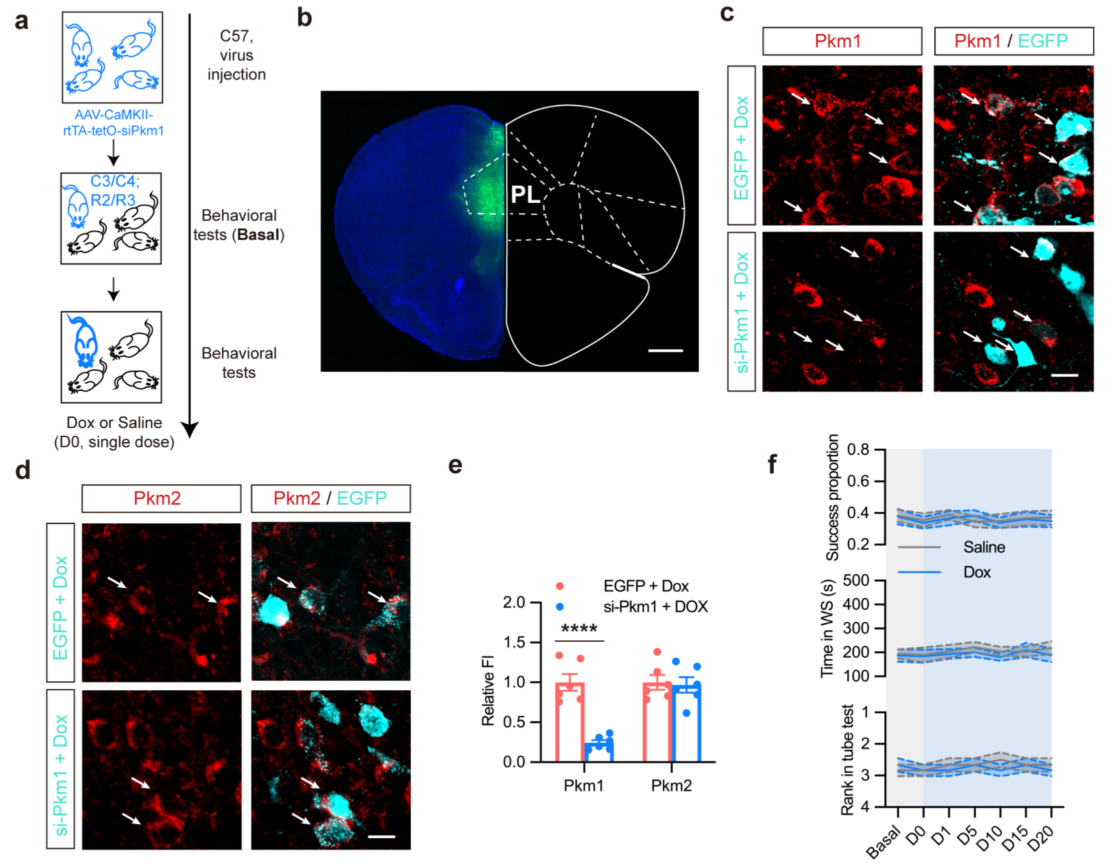

Supplementary Fig. S11. Pkm1 knockdown in PL of C3 or C4 mice does not mediate competition behavior.

a Schematic illustration of experiment.

b Representative image of EGFP fluorescence after virus injection. Bar = 500 μm.

c, d Representative immunofluorescence images with EGFP (turquoise) and antibody against Pkm1 (red) or Pkm2 (red) in PL. Bar = 50 μm.

e Quantification for relative fluorescence intensity of Pkm1 and Pkm2 in $EGFP^+$ cells of PL. *n* = 6.

f Behavioral recordings of low competitive C57 mice injected with AAV-CaMKII-rtTA-tetO-si-Pkm1-EGFP. Top: winning proportions of pellet consumption in food competition test, *n* = 18 sessions from 6 groups; middle: occupation time of warm spot in warm spot test, *n* = 6 from 6 groups; bottom: rank in tube test, *n* = 6 from 6 groups.

All data are presented as means ± SEM. For **e**, unpaired *t*-test was used. For **f**, two-way ANOVA with Dunnett's multiple-comparison test was used. ****$P < 0.0001$

Supplementary Fig. S12

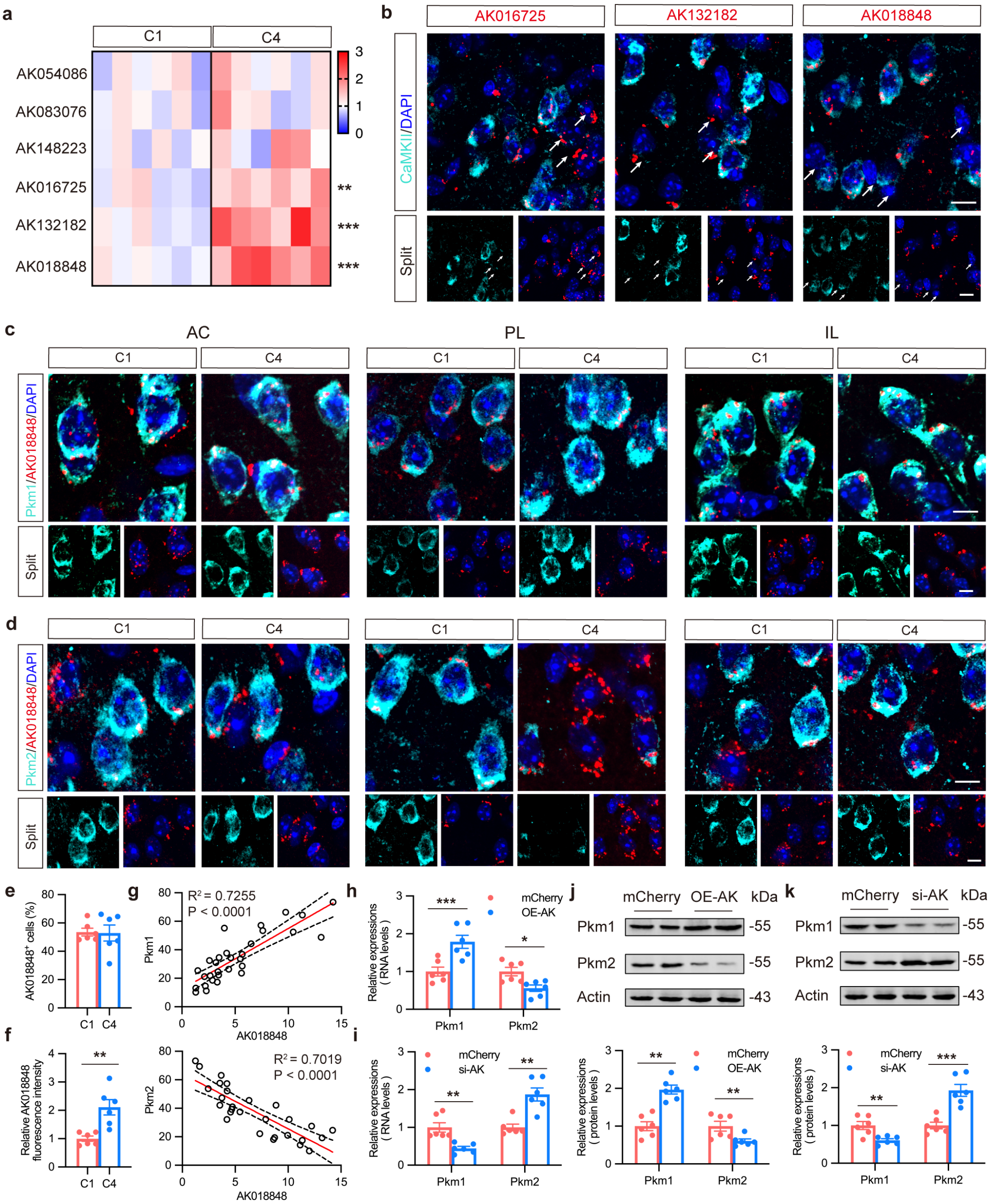

Supplementary Fig. S12. *AK018848* mediates alternative splicing of *Pkm*.

a LncRNA expression in mPFC of C1 and C4 mice detected by qPCR. *n* = 6.

b Representative double-immunofluorescence images with antibodies against CaMKII (turquoise) and FISH for *AK016725*, *AK132182* or *AK018848* (red) in PL. Bar = 30 μm.

c, d Representative double-immunofluorescence images with antibodies against Pkm1 or Pkm2 (turquoise) and FISH for *AK018848* (red) in mPFC. Bar = 30 μm.

e Lack of difference in proportion of *AK018848*$^+$ Cells. *n* = 6.

f Quantification for relative fluorescence intensity of *AK018848* in PL. *n* = 6.

g Correlation of fluorescence intensity between AK018848 and Pkm1 or Pkm2.

h, i Relative mRNA levels of Pkm1 and Pkm2 via qPCR after AAV-CMV-AK018848-mCherry or AAV-CMV-si-AK018848-mCherry application on mouse hippocampal primary neurons. *n* = 6.

j, k Representative blots (up) and quantification (down) of Pkm1 and Pkm2 protein after AAV-CMV-AK018848-mCherry or AAV-CMV-si-AK018848-mCherry was applied on mouse hippocampal primary neurons. *n* = 6.

All data are shown as means ± SEM. For **a**, **e** and **f**, paired *t*-test was used. For **h**-**k**, unpaired *t*-test was used. For **g**, simple linear regression was used. *$P < 0.05$, **$P < 0.01$, ***$P < 0.001$.

Supplementary Fig. S13

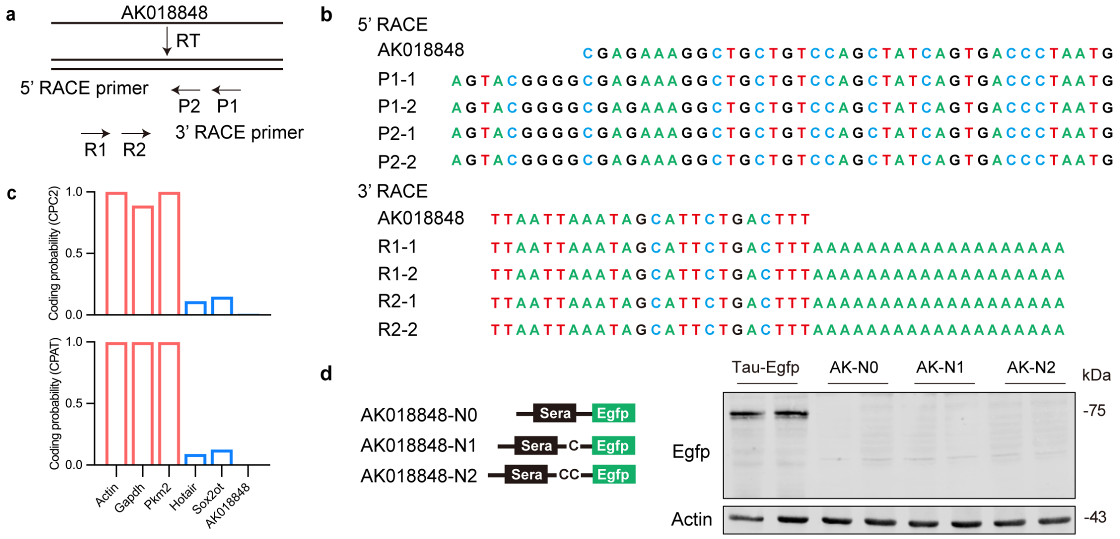

Supplementary Fig. S13. LncRNA properties of *AK018848*.

a Schematic representation of 3' and 5' rapid amplification of cDNA ends (RACE).

b Sequencing data from PCR electropherogram of 3' and 5' RACE together with BioEdit blast search alignment showed about 100% identical to the sequence of *AK018848* in UCSC.

c Coding probability of known mRNAs (Actin, Gapdh, Pkm2) or lncRNAs (*Hotair*, *Xist* and *Sox2ot*) compared to *AK018848*.

d Full length of *AK018848* was cloned into the vector pEGFP-N1 with N-terminal start codon ATG and C-terminal EGFP tag. These plasmids and Tau-EGFP (positive control) plasmid were separately transfected into N2a cells. After 72 hours, EGFP-tagged proteins were detected by immunoblot assays. *n* = 3.

Supplementary Fig. S14

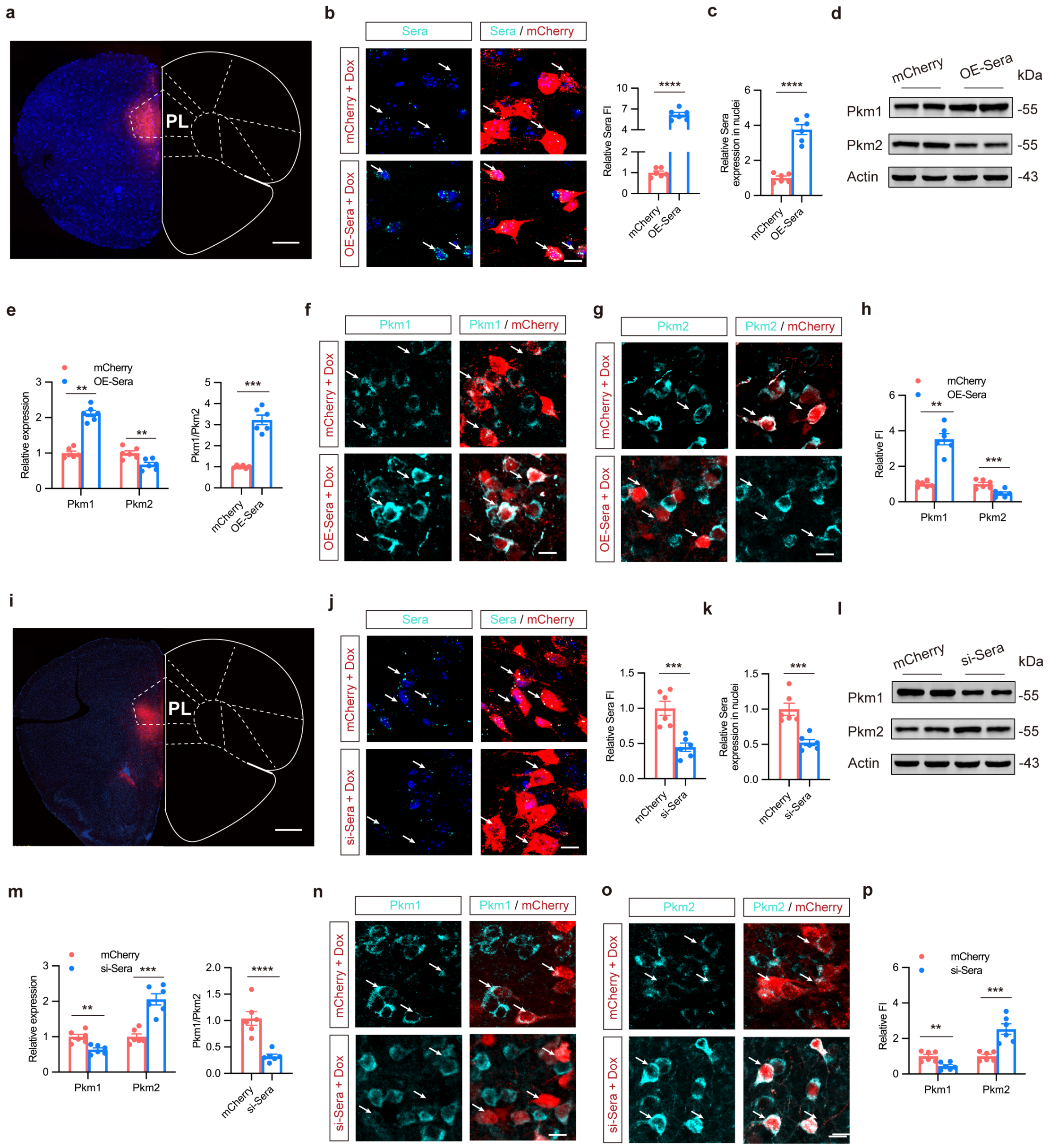

Supplementary Fig. S14. *Sera* mediation regulates expression of Pkm1 and Pkm2

a Representative image of mCherry fluorescence after virus injection. Bar = 500 μm.

b Representative immunofluorescence images with mCherry and FISH for Sera and quantification. Bar = 50 μm. n = 6.

c Relative Sera expression in nuclei via qPCR. n = 6.

d, e Representative blots and quantification of Pkm1 and Pkm2 protein after virus injection in PL. n = 6.

f-h Representative immunofluorescence images with antibodies against Pkm1 (turquoise) or Pkm2 (turquoise) in PL and quantification. Bar = 50 μm.

i Representative image of mCherry fluorescence after virus injection. Bar = 500 μm.

j Representative immunofluorescence images with mCherry and FISH for Sera and quantification. Bar = 50μm. *n* = 6.

k Relative Sera expression in nuclei via qPCR. *n* = 6.

l, m Representative blots and quantification of Pkm1 and Pkm2 protein after virus injection in PL. *n* = 6.

n-p Representative immunofluorescence images with antibodies against Pkm1 (turquoise) or Pkm2 (turquoise) in PL and quantification. Bar = 50 μm.

All data are presented as means ± SEM and analyzed by unpaired *t*-test. $^{**}P < 0.01$, $^{***}P < 0.001$, and $^{****}P < 0.0001$.

Supplementary Fig. S15

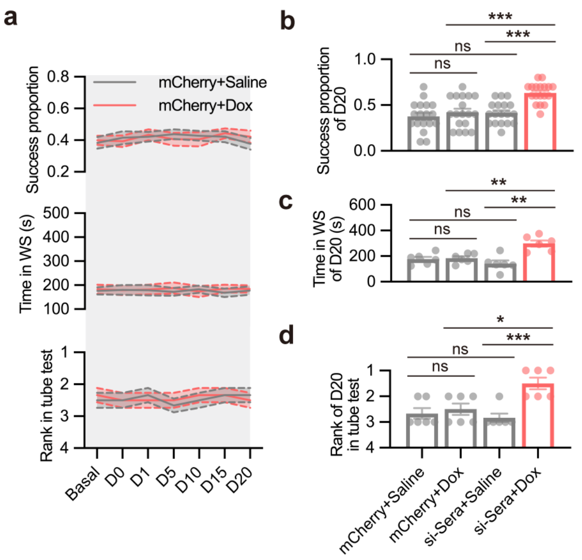

Supplementary Fig. S15. Dox treatment and the control virus did not affect social competition and rank in low competitive mice

a Behavioral recordings of low competitive C57 mice injected with AAV-CaMKII-rtTA-tetO-siRNA-mCherry. Top: winning proportions of pellet consumption in food competition test, *n* = 18 sessions from 6 groups; middle: occupation time of warm spot in warm spot test, *n* = 6 from 6 groups; bottom: rank in tube test, *n* = 6 from 6 groups.

b Winning proportions of pellet consumption in food competition test on day 20, *n* = 18 sessions from 6 groups.

c Occupation time of warm spot in warm spot test on day 20, *n* = 6 from 6 groups.

d Rank in tube test on day 20, *n* = 6 from 6 groups.

All data are presented as means ± SEM. For **a**, two-way ANOVA with Bonferroni analysis was used. For **b-d**, two-way ANOVA with Dunnett's multiple-comparison test was used. *$P < 0.05$, **$P < 0.01$, ***$P < 0.001$.

Supplementary Fig. S16

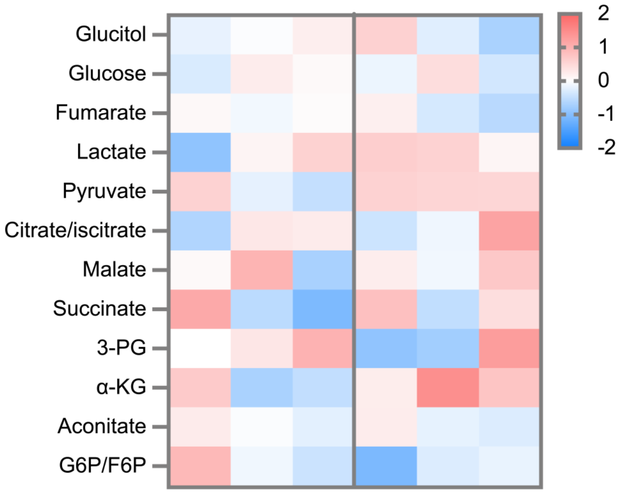

Supplementary Fig. S16. Metabolomics data show no difference in glycolysis between C1 and C4 mice.

Relative levels of key glycolysis metabolites in mPFC detected by metabolomics. n = 3.

Supplementary Fig. S17

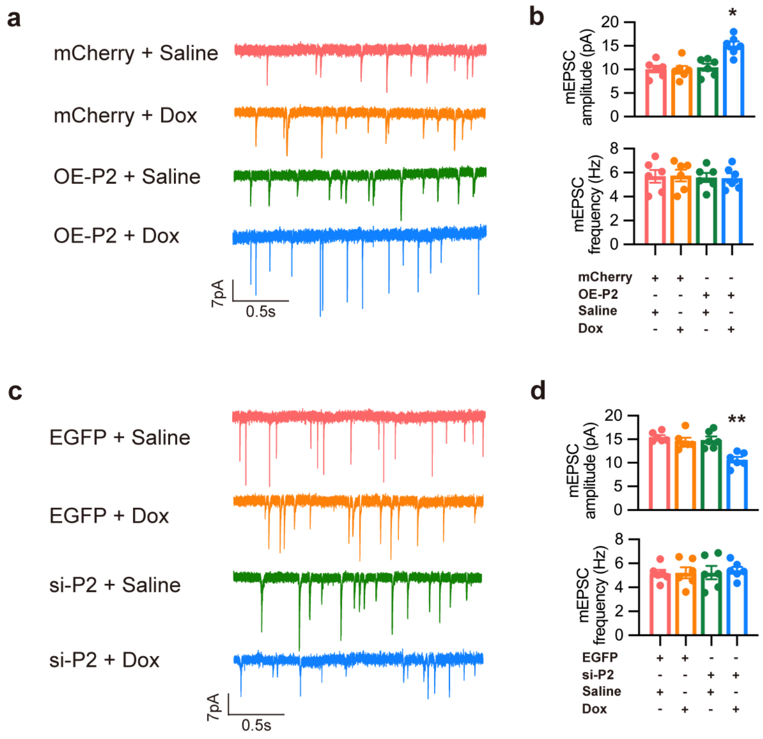

Supplementary Fig. S17. mEPSCs of PL neurons were changed after Pkm2 mediation.

a Representative traces of mEPSCs from PL neurons of WT C3 or C4 mice overexpressed with tet-on Pkm2 or mCherry, with or without Dox application.

b the mean mEPSC amplitude and frequency were analyzed. *n* = 30 neurons from 6 mice for each group.

c Representative traces of mEPSCs from PL neurons of WT C1 or C2 mice overexpressed with tet-on si-Pkm2 or EGFP, with or without Dox application.

d the mean mEPSC amplitude and frequency were analyzed. *n* = 30 neurons from 6 mice for each group.

All data are presented as means ± SEM and analyzed by two-way ANOVA with Dunnett's multiple-comparison test. **P* < 0.05, ***P* < 0.01.

Supplementary Fig. S18

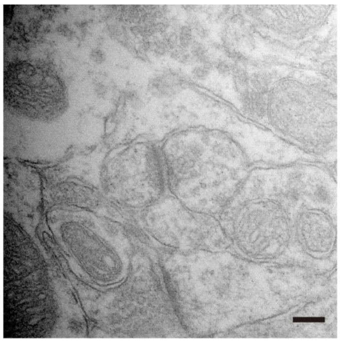

Supplementary Fig. S18. Negative control of immunoelectron microscopy. Representative immunoelectron microscope image for mouse mPFC staining with negative control. Bar = 100 nm.

Supplementary Fig. S19

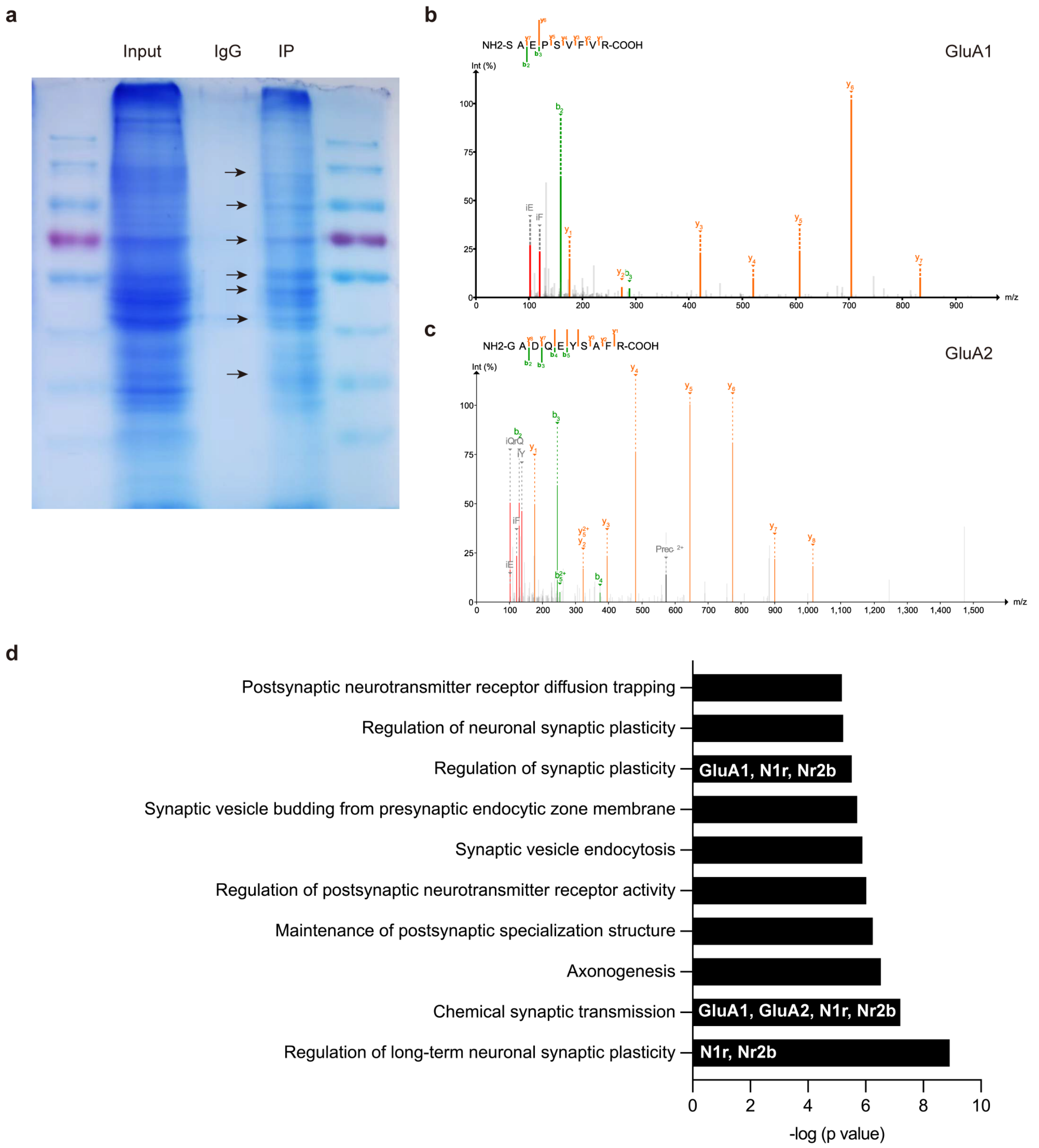

Supplementary Fig. S19. Pkm2-binding proteins detected by mass spectrum.

a Commassie blue gel staining. Input: proteins of extracts without IP. IgG and IP: Co-IP of proteins from mPFC with nonspecific IgG or anti-Pkm2.

b, c Base peak chromatogram of GluA1 and GluA2 in mass spectrum analysis.

d The main category terms of GO biological process analysis of Pkm2-binding proteins.

Supplementary Fig. S20

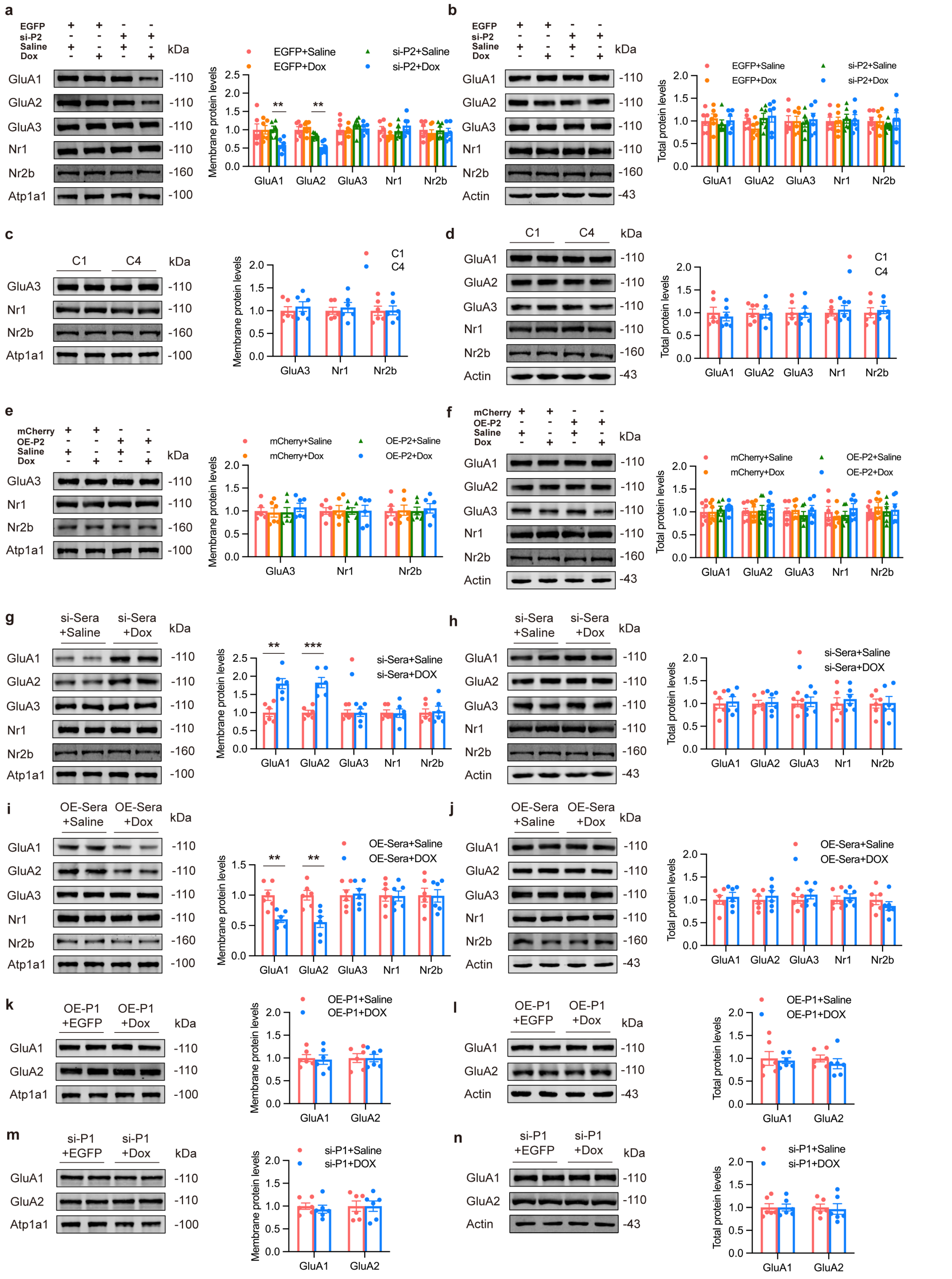

Supplementary Fig. S20. *Sera* and Pkm2 mediates membrane GluA1 and GluA2 levels

Representative blots (left) and quantification (right) of membrane (**a**, **c**, **e**, **g**, **i**, **k** and **m**) and total (**b**, **d**, **f**, **h**, **j**, **l** and **n**) AMPA-R and NMDA-R levels. All data are presented as means ± SEM. For **a**, **b**, **e** and **f**, two-way ANOVA with Dunnett's multiple-comparison test was used. For **c**, **d** and **g-n**, paired *t*-test was used. $^{**}P < 0.01$, $^{***}P < 0.001$.

Supplementary Fig. S21

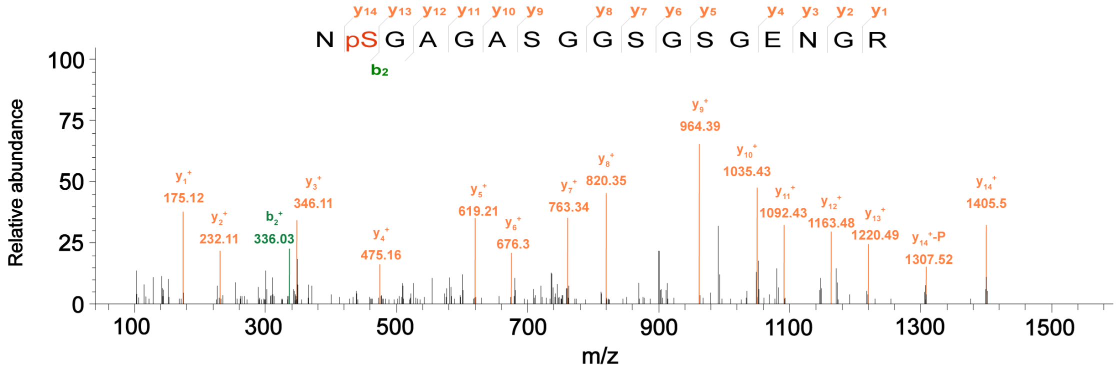

Supplementary Fig. S21. Direct phosphorylation of GluA1 at Ser845 by Pkm2.

Phosphorylated GluA1 by Pkm2 analyzed by mass spectrometry (MS). Note that MS analysis of fragment of GluA1 treated with PEP/Pkm2 matches to the peptide NSGAGASGGSGSGENGR of GluA1, suggesting that GluA1 Ser845 was phosphorylated.

Supplementary Fig. S22

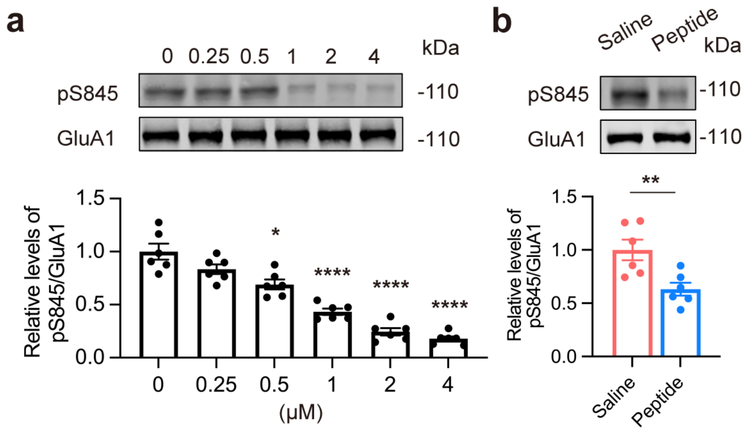

Figure S22. Validation of peptide against GluA1 S845 phosphorylation.
a Representative blots (up) and quantification (down) of phosphorylated GluA1 S845 after peptide was transfected to mouse primary hippocampal neuron. *n* = 6.
b Representative blots (left) and quantification (right) of phosphorylated GluA1 S845 in mPFC after peptide was applied to mice.
All data are presented as means ± SEM. For **a**, two-way ANOVA with Dunnett's multiple-comparison test was used. For **b**, paired *t*-test was used. $^*P < 0.05$, $^{**}P < 0.01$, $^{****}P < 0.0001$.

Supplementary Tables

Supplementary Table S1. Antibodies used in immunofluorescence and Western blot

Antibodies	Source	Identifier
Anti-CaMKII alpha antibody [6G9]	Abcam	Cat# ab22609
Anti-c-fos antibody [EPR20769]	Abcam	Cat# ab214672
Anti-Egfp antibody [F56-6A1.2.3]	Abcam	Cat# ab184601
Anti-Gad67 antibody [F-6]	Santa Cruz	Cat# sc-28376
Anti-Pkm1 antibody [D30G6]	Cell signaling	Cat# 7067
Anti-Pkm1 antibody	ABclonal	Cat# A18800
Anti-Pkm2 antibody [D78A4]	Cell signaling	Cat# 4053
Anti-Pkm2 antibody	ABclonal	Cat# A18799
Goat anti-Mouse IgG (H+L) Secondary Antibody, Alexa Fluor™ 488	Invitrogen	Cat# A11001
Goat anti-Rabbit IgG (H+L) Secondary Antibody, Alexa Fluor™ 594	Invitrogen	Cat# A11012
Beta Actin Monoclonal antibody	Proteintech	Cat# 66009-1-Ig
Atp1a1 Polyclonal antibody	Proteintech	Cat# 14418-1-AP
Glua1 Monoclonal antibody	Proteintech	Cat# 67642-1-Ig
Glua2 Polyclonal antibody	Proteintech	Cat# 11994-1-AP
Glua3 Polyclonal antibody	Proteintech	Cat# 29588-1-AP
Nr1 Polyclonal antibody	Proteintech	Cat# 27676-1-AP
Nr2b Polyclonal antibody	Proteintech	Cat# 21920-1-AP

Supplementary Table S2. Primers used in qPCR, RACE and RIP

Method	Gene or sequence	Primer (5' to 3')
qPCR		
	Pkm1 (or E9)	F: TGCAGCACCTGATAGCTCGG<br>R: GTGAGCACTCCTGCCAGACTC
	Pkm2 (or E10)	F: AGCACCTGATTGCCCGAGA<br>R: TGAGCACTCCTGCCAGACTTG
	Fbxo11 E1	F: GCAGCACAACTGAATTCCTTCC<br>R: CAACTGACCTTGTGTGTCCATTTT
	Kif21a e19	F: GACTCAAACAGACCGAAATCACC<br>R: CCATCTAGATCTTGCAGTGCGT
	Mapk8ip3 e4	F: GGAGCAGTACCGCCAGGTTC<br>R: TGGGGCTCAGCTGCTTGTAC
	Mapk8ip3 e18	F: TGAGAAGAAGAAGGCAAAGGAAAC<br>R: TGGGCATTGCAGACTGTGAAC
	Mapk8ip3 e19	F: CCCCTGAGTGAGACCCTGTCC<br>R: GGCAAAAGCTGCAAGTTGAGAG
	Mapt e19	F: CAGGGTTTGTGATCAGGCTCC<br>R: TGTCTATGAGGAGCAGCGGG
	Syne1 e118	F: CACCACTTCTAAACGTTCCTCTCTG<br>R: TTGATACCAGTTCTTGGAGTGAGG
	Tanc2 e12	F: GACCAGTTTACAGGAAATTACCAAG<br>R: TGTAGTATTGTCCATCTTGCCAT
	Sera	F: CCCCAGAAAGTGGCTTGC<br>R: AAAAGTCAGTGTTGATGCTAAGTTC
	AK054086	F: CCTGACCCGTGAGGCAAGAT<br>R: CTACTTAGAAGGTGAAAGGGCTGG
	AK083076	F: TGGGTGAGGACCCAGAACTT<br>R: TGGTTCTTTGCTGGCCGAA
	AK148223	F: GCATTGTTGGCTAACTTTCTTGG<br>R: GGGGCTTTTTTTTCTTTCAGTT
	AK016725	F: GCGGCATTCCCAGAGTCG<br>R: AGCGGCCAATAGGCGTG
	AK132182	F: GGTAGAGGGTATCGCTCGC<br>R: ACTGGGTCTGCTCACTTGC
	Actin	F: GAGACCTTCAACACCCCAGC<br>R: GGAGAGCATAGCCCTCGTAGAT
RIP		
	E10(11)	F: TACCACTTGCAGCTATTCGAGG<br>R: GGAGAGGTGTCAACTGAGCGTG
RACE		
	3' AP	ReverTra:<br>GGCCACGCGTCGACTAGTACTTTT

	TTTTTTTTTTTTTTTTTTVN
3’ RACE R1	AGAAAGGCTGCTGTCCAGCT
3’ RACE R2	CCCCAGAAAGTGGCTTGC
5’ RACE P1	AAAAGTCAGTGTTGATGCTAAGTTC
5’ RACE P2	GCAAGCCACTTTCTGGGG

Supplementary Table S3. siRNA sequences and primers used for constructing overexpression plasmids

Method	Target	Sequences or primer (5' to 3')
siRNA		
	Pkm1	CCGTCTGCTGTTTGAAGAG
	Pkm2	TCTACCACTTGCAGCTATT
	Sera	GACCCTAATGAGAAGCCAA
Overexpression		
	Pkm1	Fragment1:<br>F: ATGCCGAAGCCACACAG<br>R: AACAGCAGACGGTGGAACAT<br>Fragment2:<br>F: TGAAGAGCTTGTGCGAGCCT<br>R: TCAAGGTACAGGCACTACACGCAT
	Pkm2	Fragment1:<br>F: ATGCCGAAGCCACACAG<br>R: GTGGGGTCGCTGGTAATG<br>Fragment2:<br>F: AGAAGCTGCCGCCGTGGG<br>R: TCAAGGTACAGGCACTACACGCAT
	Sera	F: CGAGAAAGGCTGCTGTCC<br>R: AAAGTCAGAATGCTATTTAATTAAG
